# Supplementary material for: Identification of outcomes to inform the development of a core outcome set for surgical innovation: a targeted review of case studies of novel surgical devices
Source: BMJ Open. 2022 Apr 28;12(4):e056003. doi: 10.1136/bmjopen-2021-056003 (PMC9058790; doi:10.1136/bmjopen-2021-056003)
Supplement: Supplementary data [file bmjopen-2021-056003supp001.pdf]

Search strategy

All searches were performed in PubMed in July 2018.

Searches were performed by entering the device trade name into the search box with no other filters or limits used. This created and ran an automatically generated search query within the PubMed database. Two examples of the automatically generated search query for two of the device case studies are shown here:

Accolade II

"Accolade"[All Fields] AND "II"[All Fields]

Braxon

"Braxon"[All Fields]
