# Supplementary material for: Identification of outcomes to inform the development of a core outcome set for surgical innovation: a targeted review of case studies of novel surgical devices
Source: BMJ Open. 2022 Apr 28;12(4):e056003. doi: 10.1136/bmjopen-2021-056003 (PMC9058790; doi:10.1136/bmjopen-2021-056003)
Supplement: Supplementary data [file bmjopen-2021-056003supp002.pdf]

Publications included in the review (n=128)

1. Afzal, M.R., et al., *Techniques for successful early retrieval of the Micra transcatheter pacing system: A worldwide experience.* Heart Rhythm, 2018. **15**(6): p. 841-846.
2. Ahmed, F.Z., et al., *Totally Leadless Dual-Device Implantation for Combined Spontaneous Ventricular Tachycardia Defibrillation and Pacemaker Function: A First Report.* Can J Cardiol, 2017. **33**(8): p. 1066.e5-1066.e7.
3. Alicuben, E.T., et al., *Worldwide Experience with Erosion of the Magnetic Sphincter Augmentation Device.* J Gastrointest Surg, 2018. **22**(8): p. 1442-1447.
4. Ascherman, J.A., et al., *Carbon Dioxide-Based versus Saline Tissue Expansion for Breast Reconstruction: Results of the XPAND Prospective, Randomized Clinical Trial.* Plast Reconstr Surg, 2016. **138**(6): p. 1161-1170.
5. Asti, E., et al., *Longitudinal comparison of quality of life in patients undergoing laparoscopic Toupet fundoplication versus magnetic sphincter augmentation: Observational cohort study with propensity score analysis.* Medicine (Baltimore), 2016. **95**(30): p. e4366.
6. Asti, E., et al., *Removal of the Magnetic Sphincter Augmentation Device: Surgical Technique and Results of a Single-center Cohort Study.* Ann Surg, 2017. **265**(5): p. 941-945.
7. Bardoli, A.D., W.S.J. Taylor, and W. Mahmalji, *Can the UroLift prostatic implant device treat the symptoms of benign prostatic hypertrophy, avoid sexual dysfunction and reduce hospital TURP waiting times? A single centre, single surgeon experience and review of the literature.* Aging Male, 2017. **20**(3): p. 192-197.
8. Bauer, M., et al., *Endoluminal perforation of a magnetic antireflux device.* Surg Endosc, 2015. **29**(12): p. 3806-10.
9. Bell, R., et al., *Laparoscopic magnetic sphincter augmentation versus double-dose proton pump inhibitors for management of moderate-to-severe regurgitation in GERD: a randomized controlled trial.* Gastrointest Endosc, 2019. **89**(1): p. 14-22.e1.
10. Berna, G., et al., *Evaluation of a novel breast reconstruction technique using the Braxon(®) acellular dermal matrix: a new muscle-sparing breast reconstruction.* ANZ J Surg, 2017. **87**(6): p. 493-498.
11. Biswal, N.C., et al., *UroLift as a surrogate for fiducial markers in IGRT planning of prostate cancer in BPH patients.* Pract Radiat Oncol, 2018. **8**(4): p. e231-e233.
12. Blom, J., et al., *Results of collagen plug occlusion of anal fistula: a multicentre study of 126 patients.* Colorectal Dis, 2014. **16**(8): p. 626-30.
13. Blumenfeld, Z., et al., *Sixty-hertz stimulation improves bradykinesia and amplifies subthalamic low-frequency oscillations.* Mov Disord, 2017. **32**(1): p. 80-88.
14. Bonavina, L., et al., *Laparoscopic sphincter augmentation device eliminates reflux symptoms and normalizes esophageal acid exposure: one- and 2-year results of a feasibility trial.* Ann Surg, 2010. **252**(5): p. 857-62.
15. Bozkurt, A., et al., *Prostatic Urethral Lift: A New Minimally Invasive Treatment for Lower Urinary Tract Symptoms Secondary to Benign Prostatic Hyperplasia.* Urol Int, 2016. **96**(2): p. 202-6.
16. Buckley, F.P., 3rd, et al., *Favorable results from a prospective evaluation of 200 patients with large hiatal hernias undergoing LINX magnetic sphincter augmentation.* Surg Endosc, 2018. **32**(4): p. 1762-1768.
17. Cantwell, A.L., et al., *Multicentre prospective crossover study of the 'prostatic urethral lift' for the treatment of lower urinary tract symptoms secondary to benign prostatic hyperplasia.* BJU Int, 2014. **113**(4): p. 615-22.
18. Chelvarajah, R., et al., *Shielded battery syndrome: a new hardware complication of deep brain stimulation.* Stereotact Funct Neurosurg, 2012. **90**(2): p. 113-7.
19. Chin, P. and P. Robertson, *Medium-term efficacy of the prostatic urethral lift.* Transl Androl Urol, 2017. **6**(Suppl 2): p. S122-s132.

20. Chinitz, L., et al., *Accelerometer-based atrioventricular synchronous pacing with a ventricular leadless pacemaker: Results from the Micra atrioventricular feasibility studies.* Heart Rhythm, 2018. **15**(9): p. 1363-1371.
21. Connell, T.F., *Patient-activated controlled expansion for breast reconstruction using controlled carbon dioxide inflation: confirmation of a feasibility study.* Plast Reconstr Surg, 2014. **134**(4): p. 503e-11e.
22. Connell, T.F., *Results from the ASPIRE study for breast reconstruction utilizing the AeroForm™ patient controlled carbon dioxide-inflated tissue expanders.* J Plast Reconstr Aesthet Surg, 2015. **68**(9): p. 1255-61.
23. de Souza, L.R., et al., *Editor's Choice - Comparison of Renal Outcomes in Patients Treated by Zenith(®) Fenestrated and Zenith(®) Abdominal Aortic Aneurysm Stent grafts in US Prospective Pivotal Trials.* Eur J Vasc Endovasc Surg, 2017. **53**(5): p. 648-655.
24. Desart, K., et al., *Gastroesophageal Reflux Management with the LINX® System for Gastroesophageal Reflux Disease Following Laparoscopic Sleeve Gastrectomy.* J Gastrointest Surg, 2015. **19**(10): p. 1782-6.
25. Dorsey, C., V. Chandra, and J.T. Lee, *The "terrace technique"--totally endovascular repair of a type IV thoracoabdominal aortic aneurysm.* Ann Vasc Surg, 2014. **28**(6): p. 1563.e11-6.
26. Duray, G.Z., et al., *Long-term performance of a transcatheter pacing system: 12-Month results from the Micra Transcatheter Pacing Study.* Heart Rhythm, 2017. **14**(5): p. 702-709.
27. El-Chami, M., et al., *Impact of operator experience and training strategy on procedural outcomes with leadless pacing: Insights from the Micra Transcatheter Pacing Study.* Pacing Clin Electrophysiol, 2017. **40**(7): p. 834-842.
28. England, A., et al., *Migration of fenestrated aortic stent grafts.* J Vasc Surg, 2013. **57**(6): p. 1543-52.
29. England, A., M. García-Fiñana, and R.G. McWilliams, *Multicenter retrospective investigation into migration of fenestrated aortic stent grafts.* J Vasc Surg, 2015. **62**(4): p. 884-92.
30. Farber, M.A., R. Vallabhaneni, and W.A. Marston, *"Off-the-shelf" devices for complex aortic aneurysm repair.* J Vasc Surg, 2014. **60**(3): p. 579-84.
31. Filgate, R., A. Thomas, and M. Ballal, *Treatment of foregut fistula with biologic plugs.* Surg Endosc, 2015. **29**(7): p. 2006-12.
32. Fisher, B., et al., *Battery Longevity Comparison of Two Commonly Available Dual Channel Implantable Pulse Generators Used for Subthalamic Nucleus Stimulation in Parkinson's Disease.* Stereotact Funct Neurosurg, 2018. **96**(3): p. 151-156.
33. Gallitto, E., et al., *Results of standard suprarenal fixation endografts for abdominal aortic aneurysms with neck length ⩽10 mm in high-risk patients unfit for open repair and fenestrated endograft.* J Vasc Surg, 2016. **64**(3): p. 563-570.e1.
34. Gallitto, E., et al., *Endovascular Repair of Thoracoabdominal Aortic Aneurysm in High-Surgical Risk Patients: Fenestrated and Branched Endografts.* Ann Vasc Surg, 2017. **40**: p. 170-177.
35. Gerdes, C. and J.C. Nielsen, *Retrieval of Medtronic Micra Transcatheter Pacing System after tether removal.* Europace, 2016. **18**(8): p. 1202.
36. Gonsalves, S., et al., *Assessment of the efficacy of the rectovaginal button fistula plug for the treatment of ileal pouch-vaginal and rectovaginal fistulas.* Dis Colon Rectum, 2009. **52**(11): p. 1877-81.
37. Gratzke, C., et al., *Prostatic urethral lift vs transurethral resection of the prostate: 2-year results of the BPH6 prospective, multicentre, randomized study.* BJU Int, 2017. **119**(5): p. 767-775.
38. Greenberg, R.K., et al., *Intermediate results of a United States multicenter trial of fenestrated endograft repair for juxtarenal abdominal aortic aneurysms.* J Vasc Surg, 2009. **50**(4): p. 730-737.e1.

39. Grubman, E., et al., *To retrieve, or not to retrieve: System revisions with the Micra transcatheter pacemaker.* Heart Rhythm, 2017. **14**(12): p. 1801-1806.
40. Halak, M., M.A. Goodman, and S.R. Baker, *The fate of target visceral vessels after fenestrated endovascular aortic repair--general considerations and mid-term results.* Eur J Vasc Endovasc Surg, 2006. **32**(2): p. 124-8.
41. Harvey, J.R., et al., *Safety and feasibility of breast lesion localization using magnetic seeds (Magseed): a multi-centre, open-label cohort study.* Breast Cancer Res Treat, 2018. **169**(3): p. 531-536.
42. Hawasli, A., A. Phillips, and M. Tarboush, *Laparoscopic management of reflux after Roux-en-Y gastric bypass using the LINX system and repair of hiatal hernia: a case report.* Surg Obes Relat Dis, 2016. **12**(5): p. e51-e54.
43. Hawasli, A., M. Tarakji, and M. Tarboush, *Laparoscopic management of severe reflux after sleeve gastrectomy using the LINX(®) system: Technique and one year follow up case report.* Int J Surg Case Rep, 2017. **30**: p. 148-151.
44. Helmers, A.K., et al., *Comparison of the Battery Life of Nonrechargeable Generators for Deep Brain Stimulation.* Neuromodulation, 2018. **21**(6): p. 593-596.
45. Hemm, S., et al., *Contact position analysis of deep brain stimulation electrodes on post-operative CT images.* Acta Neurochir (Wien), 2009. **151**(7): p. 823-9; discussion 829.
46. Henderson, F., Jr. and I. Takacs, *Rapid and Minimally Traumatic Replacement of Stimulator Extension Cables: Technical Note on a Novel Use for Sternal Wire.* World Neurosurg, 2017. **97**: p. 547-550.
47. Herron, J.A., et al., *Chronic electrocorticography for sensing movement intention and closed-loop deep brain stimulation with wearable sensors in an essential tremor patient.* J Neurosurg, 2017. **127**(3): p. 580-587.
48. Holm, N., A. Müller, and R. Zbinden, *Complications with the MICRA TPS Pacemaker System: Persistent Complete Heart Block and Late Capture Failure.* Pacing Clin Electrophysiol, 2017. **40**(4): p. 455-456.
49. Iqbal, F.M., A. Bhatnagar, and R. Vidya, *Host Integration of an Acellular Dermal Matrix: Braxon Mesh in Breast Reconstruction.* Clin Breast Cancer, 2016. **16**(6): p. e209-e211.
50. Iqbal, M., et al., *Low power HOLEP after failed urolift: A case report using 50 Watt laser.* Urol Case Rep, 2018. **16**: p. 114-115.
51. Issa, K., et al., *Radiographic fit and fill analysis of a new second-generation proximally coated cementless stem compared to its predicate design.* J Arthroplasty, 2014. **29**(1): p. 192-8.
52. Jafferbhoy, S., et al., *Early multicentre experience of pre-pectoral implant based immediate breast reconstruction using Braxon(®).* Gland Surg, 2017. **6**(6): p. 682-688.
53. Kalder, J., et al., *Anatomic changes of target vessels after fenestrated and branched aortic aneurysm repair.* J Cardiovasc Surg (Torino), 2014. **55**(2 Suppl 1): p. 115-21.
54. Kaminska, M., et al., *Rechargeable deep brain stimulators in the management of paediatric dystonia: well tolerated with a low complication rate.* Stereotact Funct Neurosurg, 2012. **90**(4): p. 233-9.
55. Kaminska, M., et al., *Complications of Deep Brain Stimulation (DBS) for dystonia in children - The challenges and 10 year experience in a large paediatric cohort.* Eur J Paediatr Neurol, 2017. **21**(1): p. 168-175.
56. Kascak, P. and B. Kopcan, *Fatal Injury of the Small Intestine during Retropubic Sling Placement.* Case Rep Obstet Gynecol, 2015. **2015**: p. 164545.
57. Kawasaki, A., et al., *Comparing the risk of urethrolysis for the treatment of voiding dysfunction between two retropubic mesh slings: a case-control study.* Int Urogynecol J, 2013. **24**(4): p. 589-94.
58. Keehn, A., et al., *UroLift in Place of Fiducial Markers for Patients With Benign Prostatic Hyperplasia Undergoing External Beam Radiation Therapy.* Urology, 2017. **104**: p. 230-234.

59. Kelley, K. and J. Kim, *Human Factors Validation of the AeroForm Tissue Expander System for Breast Reconstruction.* Plast Surg Nurs, 2017. **37**(3): p. 100-102.
60. Kerwin, S.A., M.J. Mayotte, and C.C. Gornick, *Transcatheter pacemaker implantation in a patient with a bioprosthetic tricuspid valve.* J Interv Card Electrophysiol, 2015. **44**(1): p. 89-90.
61. Kitagawa, A., et al., *Zenith p-branch standard fenestrated endovascular graft for juxtarenal abdominal aortic aneurysms.* J Vasc Surg, 2013. **58**(2): p. 291-300.
62. Kypta, A., et al., *Dawn of a new era: the completely interventionally treated patient.* BMJ Case Rep, 2016. **2016**.
63. Lala, S., et al., *Superior mesenteric artery outcomes after fenestrated endovascular aortic aneurysm repair.* J Vasc Surg, 2016. **64**(3): p. 692-7.
64. Lin, J.-P., et al., *Bilateral globus pallidus internus deep brain stimulation for dyskinetic cerebral palsy supports success of cochlear implantation in a 5-year old ex-24 week preterm twin with absent cerebellar hemispheres.* European Journal of Paediatric Neurology, 2017. **21**(1): p. 202-213.
65. Lipham, J.C., et al., *The LINX® reflux management system: confirmed safety and efficacy now at 4 years.* Surg Endosc, 2012. **26**(10): p. 2944-9.
66. Lloyd, M., et al., *Rate adaptive pacing in an intracardiac pacemaker.* Heart Rhythm, 2017. **14**(2): p. 200-205.
67. Louie, B.E., et al., *Objective Evidence of Reflux Control After Magnetic Sphincter Augmentation: One Year Results From a Post Approval Study.* Ann Surg, 2019. **270**(2): p. 302-308.
68. Lyon, J.W., et al., *First experience with the use of a collagen fistula plug to treat enterocutaneous fistulas.* J Vasc Interv Radiol, 2013. **24**(10): p. 1559-65.
69. Madsen, A.M., et al., *A cohort study comparing a single-incision sling with a retropubic midurethral sling.* Int Urogynecol J, 2014. **25**(3): p. 351-8.
70. Maling, N., et al., *Biophysical basis of subthalamic local field potentials recorded from deep brain stimulation electrodes.* J Neurophysiol, 2018. **120**(4): p. 1932-1944.
71. Maruccia, M., G. Di Taranto, and M.G. Onesti, *One-stage muscle-sparing breast reconstruction in elderly patients: A new tool for retaining excellent quality of life.* Breast J, 2018. **24**(2): p. 180-183.
72. Maruccia, M., et al., *One-stage breast reconstruction techniques in elderly patients to preserve quality of life.* Eur Rev Med Pharmacol Sci, 2016. **20**(24): p. 5058-5066.
73. McAdams, S., et al., *Holmium Laser Enucleation of the Prostate After Prostatic Urethral Lift Surgery: Feasibility and Technical Considerations from a Multi-Institutional Case Series.* J Endourol, 2017. **31**(8): p. 774-779.
74. McNicholas, T.A., et al., *Minimally invasive prostatic urethral lift: surgical technique and multinational experience.* Eur Urol, 2013. **64**(2): p. 292-9.
75. Morita, J., et al., *Retrieval of a Micra transcatheter pacing system in a heart with a preexisting lead.* Indian Pacing Electrophysiol J, 2018. **18**(5): p. 183-184.
76. Nam, D., et al., *An evaluation of proximal femur bone density in young, active patients undergoing total hip arthroplasty at one year postoperatively.* Hip international : the journal of clinical and experimental research on hip pathology and therapy, 2019. **29**(1): p. 51-57.
77. Neumann, W.J., et al., *Long term correlation of subthalamic beta band activity with motor impairment in patients with Parkinson's disease.* Clin Neurophysiol, 2017. **128**(11): p. 2286-2291.
78. Niemann, M., et al., *Longevity of Implantable Pulse Generators in Bilateral Deep Brain Stimulation for Movement Disorders.* Neuromodulation, 2018. **21**(6): p. 597-603.
79. Ondo, W.G., C. Meilak, and K.D. Vuong, *Predictors of battery life for the Activa Soletra 7426 Neurostimulator.* Parkinsonism Relat Disord, 2007. **13**(4): p. 240-2.

80. Onesti, M.G., et al., *Clinical, histological, and ultrasound follow-up of breast reconstruction with one-stage muscle-sparing "wrap" technique: A single-center experience.* J Plast Reconstr Aesthet Surg, 2017. **70**(11): p. 1527-1536.
81. O'Rawe, J.A., et al., *Integrating precision medicine in the study and clinical treatment of a severely mentally ill person.* PeerJ, 2013. **1**: p. e177.
82. Parmar, A.D., et al., *Two-Stage Explantation of a Magnetic Lower Esophageal Sphincter Augmentation Device Due to Esophageal Erosion.* J Laparoendosc Adv Surg Tech A, 2017. **27**(8): p. 829-833.
83. Perez, J., et al., *Rechargeable or Nonrechargeable Deep Brain Stimulation in Dystonia: A Cost Analysis.* Neuromodulation, 2017. **20**(3): p. 243-247.
84. Piccini, J.P., et al., *Long-term outcomes in leadless Micra transcatheter pacemakers with elevated thresholds at implantation: Results from the Micra Transcatheter Pacing System Global Clinical Trial.* Heart Rhythm, 2017. **14**(5): p. 685-691.
85. Pollo, C., et al., *Magnetic resonance artifact induced by the electrode Activa 3389: an in vitro and in vivo study.* Acta Neurochir (Wien), 2004. **146**(2): p. 161-4.
86. Price, E.R., et al., *Initial Clinical Experience With an Inducible Magnetic Seed System for Preoperative Breast Lesion Localization.* AJR Am J Roentgenol, 2018. **210**(4): p. 913-917.
87. Quinn, E.J., et al., *Beta oscillations in freely moving Parkinson's subjects are attenuated during deep brain stimulation.* Mov Disord, 2015. **30**(13): p. 1750-8.
88. Ramirez de Noriega, F., et al., *Constant Current versus Constant Voltage Subthalamic Nucleus Deep Brain Stimulation in Parkinson's Disease.* Stereotact Funct Neurosurg, 2015. **93**(2): p. 114-121.
89. Reynolds, D., et al., *A Leadless Intracardiac Transcatheter Pacing System.* N Engl J Med, 2016. **374**(6): p. 533-41.
90. Reynolds, J.L., et al., *Magnetic sphincter augmentation with the LINX device for gastroesophageal reflux disease after U.S. Food and Drug Administration approval.* Am Surg, 2014. **80**(10): p. 1034-8.
91. Reynolds, J.L., et al., *Laparoscopic Magnetic Sphincter Augmentation vs Laparoscopic Nissen Fundoplication: A Matched-Pair Analysis of 100 Patients.* J Am Coll Surg, 2015. **221**(1): p. 123-8.
92. Ritter, P., et al., *Early performance of a miniaturized leadless cardiac pacemaker: the Micra Transcatheter Pacing Study.* Eur Heart J, 2015. **36**(37): p. 2510-9.
93. Roberts, P.R., et al., *A leadless pacemaker in the real-world setting: The Micra Transcatheter Pacing System Post-Approval Registry.* Heart Rhythm, 2017. **14**(9): p. 1375-1379.
94. Roehrborn, C.G., et al., *Five year results of the prospective randomized controlled prostatic urethral L.I.F.T. study.* Can J Urol, 2017. **24**(3): p. 8802-8813.
95. Roehrborn, C.G., et al., *Three year results of the prostatic urethral L.I.F.T. study.* Can J Urol, 2015. **22**(3): p. 7772-82.
96. Rona, K.A., et al., *Efficacy of magnetic sphincter augmentation in patients with large hiatal hernias.* Surg Endosc, 2017. **31**(5): p. 2096-2102.
97. Rona, K.A., et al., *Hiatal hernia recurrence following magnetic sphincter augmentation and posterior cruroplasty: intermediate-term outcomes.* Surg Endosc, 2018. **32**(7): p. 3374-3379.
98. Rukstalis, D., et al., *24-month durability after crossover to the prostatic urethral lift from randomised, blinded sham.* BJU Int, 2016. **118 Suppl 3**: p. 14-22.
99. Salvador, R., et al., *Esophageal Penetration of the Magnetic Sphincter Augmentation Device: History Repeats Itself.* J Laparoendosc Adv Surg Tech A, 2017. **27**(8): p. 834-838.
100. Sammartino, F., et al., *3-Tesla MRI in patients with fully implanted deep brain stimulation devices: a preliminary study in 10 patients.* J Neurosurg, 2017. **127**(4): p. 892-898.
101. Shahverdyan, R., et al., *Technical feasibility of endovascular aortoiliac aneurysm repair combining Anaconda fenestrated and Zenith iliac side-branched stent grafts.* J Vasc Surg, 2015. **61**(5): p. 1324-8.

102. Shahverdyan, R., et al., *F-EVAR does not Impair Renal Function more than Open Surgery for Juxtarenal Aortic Aneurysms: Single Centre Results.* Eur J Vasc Endovasc Surg, 2015. **50**(4): p. 432-41.
103. Sheu, E.G., et al., *A comparative trial of laparoscopic magnetic sphincter augmentation and Nissen fundoplication.* Surg Endosc, 2015. **29**(3): p. 505-9.
104. Shute, J.B., et al., *Thalamocortical network activity enables chronic tic detection in humans with Tourette syndrome.* Neuroimage Clin, 2016. **12**: p. 165-72.
105. Soejima, K., et al., *Performance of Leadless Pacemaker in Japanese Patients vs. Rest of the World - Results From a Global Clinical Trial.* Circ J, 2017. **81**(11): p. 1589-1595.
106. Soejima, K., et al., *Safety evaluation of a leadless transcatheter pacemaker for magnetic resonance imaging use.* Heart Rhythm, 2016. **13**(10): p. 2056-63.
107. Sriratanaviriyakul, N., et al., *LINX®, a novel treatment for patients with refractory asthma complicated by gastroesophageal reflux disease: a case report.* J Med Case Rep, 2016. **10**(1): p. 124.
108. Starnes, B.W., *Physician-modified endovascular grafts for the treatment of elective, symptomatic, or ruptured juxtarenal aortic aneurysms.* J Vasc Surg, 2012. **56**(3): p. 601-7.
109. Swann, N.C., et al., *Chronic multisite brain recordings from a totally implantable bidirectional neural interface: experience in 5 patients with Parkinson's disease.* J Neurosurg, 2018. **128**(2): p. 605-616.
110. Swann, N.C., et al., *Adaptive deep brain stimulation for Parkinson's disease using motor cortex sensing.* J Neural Eng, 2018. **15**(4): p. 046006.
111. Syrkin-Nikolau, J., et al., *Subthalamic neural entropy is a feature of freezing of gait in freely moving people with Parkinson's disease.* Neurobiol Dis, 2017. **108**: p. 288-297.
112. Tapping, C.R., et al., *Three-year follow-up of fenestrated thoracoabdominal stent graft bridging an endovascular thoracic stent graft and a surgical abdominal aortic graft.* J Vasc Interv Radiol, 2011. **22**(3): p. 385-90.
113. Tapping, C.R., M.W. Little, and P. Boardman, *Successful Prostatic Artery Embolization following UroLift Device Failure.* J Vasc Interv Radiol, 2017. **28**(9): p. 1275-1276.
114. Tatum, J.M., et al., *Minimal versus obligatory dissection of the diaphragmatic hiatus during magnetic sphincter augmentation surgery.* Surg Endosc, 2019. **33**(3): p. 782-788.
115. Timaran, D.E., et al., *Fenestrated endovascular aneurysm repair among octogenarians at high and standard risk for open repair.* J Vasc Surg, 2017. **66**(2): p. 354-359.
116. Timaran, D.E., et al., *Safety and effectiveness of total percutaneous access for fenestrated endovascular aortic aneurysm repair.* J Vasc Surg, 2016. **64**(4): p. 896-901.
117. Trager, M.H., et al., *Subthalamic beta oscillations are attenuated after withdrawal of chronic high frequency neurostimulation in Parkinson's disease.* Neurobiol Dis, 2016. **96**: p. 22-30.
118. Ugurlucan, F.G., et al., *Randomized trial of graft materials in transobturator tape operation: biological versus synthetic.* Int Urogynecol J, 2013. **24**(8): p. 1315-23.
119. Ultee, K.H.J., et al., *Perioperative outcome of endovascular repair for complex abdominal aortic aneurysms.* J Vasc Surg, 2017. **65**(6): p. 1567-1575.
120. Van Gompel, J.J., et al., *Anterior nuclear deep brain stimulation guided by concordant hippocampal recording.* Neurosurg Focus, 2015. **38**(6): p. E9.
121. Vemuri, C., et al., *Postapproval outcomes of juxtarenal aortic aneurysms treated with the Zenith fenestrated endovascular graft.* J Vasc Surg, 2014. **60**(2): p. 295-300.
122. Vidya, R. and S.J. Cawthorn, *Muscle-Sparing ADM-Assisted Breast Reconstruction Technique Using Complete Breast Implant Coverage: A Dual-Institute UK-Based Experience.* Breast Care (Basel), 2017. **12**(4): p. 251-254.
123. Vidya, R., et al., *Evaluation of the effectiveness of the prepectoral breast reconstruction with Braxon dermal matrix: First multicenter European report on 100 cases.* Breast J, 2017. **23**(6): p. 670-676.

124. Waln, O. and J. Jimenez-Shahed, *Rechargeable deep brain stimulation implantable pulse generators in movement disorders: patient satisfaction and conversion parameters.* Neuromodulation, 2014. **17**(5): p. 425-30; discussion 430.
125. Wang, S.K., et al., *Successful Treatment of an Infected Zenith Fenestrated Endograft Without Explantation.* Vasc Endovascular Surg, 2018. **52**(7): p. 569-572.
126. Warren, H.F., et al., *Factors influencing the outcome of magnetic sphincter augmentation for chronic gastroesophageal reflux disease.* Surg Endosc, 2018. **32**(1): p. 405-412.
127. Wooster, M., et al., *Concomitant Parallel Endografting and Fenestrated Experience in a Regional Aortic Center.* Ann Vasc Surg, 2017. **38**: p. 54-58.
128. Zeidler, K.R., et al., *AeroForm patient controlled tissue expansion and saline tissue expansion for breast reconstruction: a randomized controlled trial.* Ann Plast Surg, 2014. **72 Suppl 1**: p. S51-5.
