# Supplementary material for: Identification of outcomes to inform the development of a core outcome set for surgical innovation: a targeted review of case studies of novel surgical devices
Source: BMJ Open. 2022 Apr 28;12(4):e056003. doi: 10.1136/bmjopen-2021-056003 (PMC9058790; doi:10.1136/bmjopen-2021-056003)
Supplement: Author's manuscript [file bmjopen-2021-056003.draft_revisions.pdf]

BMJ Open is committed to open peer review. As part of this commitment we make the peer review history of every article we publish publicly available.

When an article is published we post the peer reviewers' comments and the authors' responses online. We also post the versions of the paper that were used during peer review. These are the versions that the peer review comments apply to.

The versions of the paper that follow are the versions that were submitted during the peer review process. They are not the versions of record or the final published versions. They should not be cited or distributed as the published version of this manuscript.

BMJ Open is an open access journal and the full, final, typeset and author-corrected version of record of the manuscript is available on our site with no access controls, subscription charges or pay-per-view fees (http://bmjopen.bmj.com).

If you have any questions on BMJ Open's open peer review process please email info.bmjopen@bmj.com

BMJ Open

# Identification of outcomes to inform the development of a core outcome set for surgical innovation: a targeted review of case studies of novel surgical devices



| | |
|---|---|
| Journal: | *BMJ Open* |
| Manuscript ID | bmjopen-2021-056003 |
| Article Type: | Original research |
| Date Submitted by the Author: | 30-Jul-2021 |



| | |
|---|---|
| Complete List of Authors: | Wilson, Nicholas; University of Bristol, Population Health Sciences<br>Macefield, Rhiannon; University of Bristol, Population Health Sciences<br>Hoffmann, Christin; University of Bristol<br>Edmondson, Matthew J; Royal United Hospitals Bath NHS Foundation Trust<br>Miller, Rachael Lucia; University of Bristol, Population Health Sciences; North Bristol NHS Trust, Department of Surgery<br>Kirkham, Emily N; Gloucestershire Hospitals NHS Foundation Trust<br>Blencowe, Natalie; University of Bristol, Population Health Sciences<br>McNair, Angus; University of Bristol, Population Health Sciences; North Bristol NHS Trust,  GI Surgery<br>Main, Barry; University of Bristol, Population Health Sciences; University Hospitals Bristol and Weston NHS Foundation Trust, Division of Surgery<br>Blazeby, Jane; University of Bristol, Department of Social Medicine<br>Avery, Kerry; University of Bristol, Population Health Sciences<br>Potter, Shelley; University of Bristol, Population Health Sciences; North Bristol NHS Trust, Bristol Breast Care Centre |



| | |
|---|---|
| Keywords: | STATISTICS & RESEARCH METHODS, SURGERY, PUBLIC HEALTH |


SCHOLARONE™
Manuscripts

*I, the Submitting Author has the right to grant and does grant on behalf of all authors of the Work (as defined in the below author licence), an exclusive licence and/or a non-exclusive licence for contributions from authors who are: i) UK Crown employees; ii) where BMJ has agreed a CC-BY licence shall apply, and/or iii) in accordance with the terms applicable for US Federal Government officers or employees acting as part of their official duties; on a worldwide, perpetual, irrevocable, royalty-free basis to BMJ Publishing Group Ltd ("BMJ") its licensees and where the relevant Journal is co-owned by BMJ to the co-owners of the Journal, to publish the Work in this journal and any other BMJ products and to exploit all rights, as set out in our licence.*

*The Submitting Author accepts and understands that any supply made under these terms is made by BMJ to the Submitting Author unless you are acting as an employee on behalf of your employer or a postgraduate student of an affiliated institution which is paying any applicable article publishing charge ("APC") for Open Access articles. Where the Submitting Author wishes to make the Work available on an Open Access basis (and intends to pay the relevant APC), the terms of reuse of such Open Access shall be governed by a Creative Commons licence – details of these licences and which Creative Commons licence will apply to this Work are set out in our licence referred to above.*

*Other than as permitted in any relevant BMJ Author's Self Archiving Policies, I confirm this Work has not been accepted for publication elsewhere, is not being considered for publication elsewhere and does not duplicate material already published. I confirm all authors consent to publication of this Work and authorise the granting of this licence.*

For peer review only

# Identification of outcomes to inform the development of a core outcome set for surgical innovation: a targeted review of case studies of novel surgical devices

Nicholas Wilson PhD[1]*, Rhiannon C Macefield PhD[1]*, Christin Hoffmann PhD[1], Matthew J Edmondson[2], Rachael L Miller[1,3] MRes, Emily N Kirkham[4], Natalie S Blencowe PhD[1,5], Angus GK McNair PhD[1,3], Barry G Main PhD [1,5], Jane M Blazeby MD[1,5], Kerry NL Avery PhD[1]**, Shelley Potter PhD[1,6]**

*joint first authors
**joint senior authors

**Affiliations:**

[1] National Institute for Health Research Bristol Biomedical Research Centre, Bristol Centre for Surgical Research, Bristol Medical School: Population Health Sciences, University of Bristol, 39 Whatley Road, Clifton, Bristol, BS8 2PS, UK

[2]Intensive Care, Royal United Hospitals Bath NHS Foundation Trust, BA1 3NG

[3] Department of Surgery, North Bristol NHS Trust, Southmead Road, Bristol, BS10 5NB

[4] Gloucestershire Hospitals NHS Foundation Trust, Great Western Road, Gloucester, GL1 3NN

[5] Division of Surgery, Bristol Royal Infirmary, University Hospitals Bristol and Weston NHS Foundation Trust, Bristol, BS2 8HW

[6] Bristol Breast Care Centre, North Bristol NHS Trust, Southmead Road, Bristol, BS10 5NB

**Corresponding author:**

Dr Rhiannon C Macefield, Bristol Centre for Surgical Research, University of Bristol, Canynge Hall, 39 Whatley Road, Clifton, Bristol, BS8 2PS, UK. Email: r.macefield@bristol.ac.uk

For peer review only



For peer review only - http://bmjopen.bmj.com/site/about/guidelines.xhtml

**Abstract**

**Objective:** Outcome selection and reporting in studies of novel surgical procedures and devices lacks standardisation, hindering safe and effective evaluation. A core outcome set (COS) to measure and report in all studies of surgical innovation is needed. We explored outcomes in a specific sample of innovative surgical device case studies to identify outcome domains specifically relevant to innovation to inform development of a COS.

**Design:** A targeted review of 11 purposive-selected case studies of innovative surgical devices.

**Methods:** Electronic database searches identified publications reporting the introduction and evaluation of each device. Outcomes were extracted and categorised into domains until no new domains were conceptualised. Outcomes specifically relevant to evaluating innovation were further scrutinised.

**Results:** 112 relevant publications were identified, and 5,926 outcomes extracted. Heterogeneity in study type, outcome selection and reporting was observed across surgical devices. Categorisation of outcomes was performed for 2689 (45.4%) outcomes into five broad outcome domains. Outcomes considered key to the evaluation of innovation (n=66; 2.5%) were further categorised as surgeon/operator experience (n=40; 1.5%), unanticipated events (n=15, 0.6%), and modifications (n=11; 0.4%).

**Conclusion:** Outcome domains unique to evaluating innovation have been identified. This study was the first of multiple data sources to inform the development of a COS to measure and report in all studies evaluating novel surgical procedures/devices.

For peer review only

**Article summary**

**Strengths and limitations of this study**

- A purposive sample of surgical device case studies was selected to ensure broad representation across surgical specialties and disease areas.
- Studies were chosen to reflect the breadth of surgical innovation.
- A broad definition of an outcome was adopted to ensure that all information potentially relevant to evaluating innovation was captured.
- This review was limited by searching for relevant publications using device trade name only and restricting to English language publications.

For peer review only



For peer review only - http://bmjopen.bmj.com/site/about/guidelines.xhtml

## Introduction

The introduction and evaluation of innovative surgical procedures and devices is less regulated and standardised than that required for new pharmaceuticals. This can cause patient harm, as demonstrated by examples such as the introduction of metal-on-metal hip replacements and vaginal mesh implants [1, 2]. One particular challenge for the evaluation of surgical innovation is the lack of standardisation in the selection, measurement and reporting of outcomes in studies of new procedures and devices, limiting data synthesis and the ability to accurately compare and contrast device efficacy [3]. Decisions regarding the choice of outcomes are currently made by study investigators. Outcomes may be selected *a priori* (based on hypothesised efficacy) or *post hoc* (based on statistical significance), rather than being selected because they are meaningful to patients or valuable to surgeon-innovators and other stakeholders looking to adopt the procedure. These issues risk the potential for introducing outcome reporting bias and inconsistent and heterogenous outcome selection which, when combined with thwarted data synthesis, may delay identification of the benefits and harms of new interventions [4].

A potential solution to this problem is to develop and use a core outcome set (COS) to evaluate innovative surgical procedures/devices. A core outcome set is an agreed set of outcomes to be measured and reported, as a minimum, in all studies in a particular area [5]. Use of COS have been shown to be effective at improving the quality and consistency of outcome selection and reporting in comparative effectiveness research, for example, randomised controlled trials (RCTs) [6]. Methods for developing COS for effectiveness studies for particular diseases or conditions are well-established. This includes the identification of a 'long list' of all potential outcomes to inform the consensus process and reduce to a core set, typically by undertaking systematic reviews of RCTs in the specific disease or condition to identify outcomes of interest [5]. However, such methods are unlikely to identify outcomes of specific relevance to surgical innovation. Furthermore, important 'drivers' of innovation may not be recognised and reported as outcomes in the traditional sense. Novel approaches are needed. We hypothesised that publications reporting the introduction and evaluation of new surgical devices may generate specific insights into outcome selection and reporting in surgical innovation and potentially serve as one of several data sources to inform the development of a future COS.

The aim of this study was to i) examine outcome selection and reporting in a purposive sample of case studies of known innovative surgical devices, ii) to categorise outcomes into domains; identifying outcomes specifically relevant to innovation and iii) conceptualise innovation domains for inclusion in a later Delphi consensus process to develop a COS [7, 8]. This study served as the first of multiple data sources to conceptualise outcome domains for inclusion in the COS [7].



For peer review only - http://bmjopen.bmj.com/site/about/guidelines.xhtml

**Methods**

A targeted review of purposively-selected case studies of innovative surgical devices was undertaken.

*Selection of case studies*

Specialists from a range of medical disciplines (orthopaedic surgery, neurosurgery, plastic surgery, gynaecology, gastrointestinal surgery, breast surgery, cardiology, urology, vascular surgery, maxillofacial surgery and ophthalmology) known to the study team were contacted and asked to identify innovative surgical devices developed within the last decade. A purposive sample of the identified devices were selected by the study team to ensure broad representation across the surgical specialties and disease areas. Devices were further selected based on the "degree of novelty" of the innovation judged by the study team to ensure that case studies reflected the breadth of the surgical innovation lifecycle. Case studies were categorised as 'wholly innovative' (a device representing a completely new approach to solving a clinical problem); 'partially innovative' (a device broadly similar in function to one already in use but differing in at least one significant way); 'reinvented' (a modification of a device or technique that had been previously been used but that was abandoned due to complications) or 'predicate' (a device introduced via the U.S. Food and Drug Administration (FDA) predicate 510(k) pathway on the basis of equivalence [9]).

*Identification and selection of publications*

Electronic searches were performed in PubMed to identify publications reporting the introduction and evaluation of each device in July 2018. Searches were performed using the tradename of the device as a text word. No publication date limits were applied. Search results were imported into Excel. Records were screened for eligibility by one reviewer with 10 per cent independently checked for eligibility by a second reviewer. Included were primary studies reporting the use of the device at any stage of development from first in human up to and including RCTs. Excluded were studies involving animals and publications in non-English languages. Letters and conference proceedings were also excluded due to lack of detail to extract comprehensive information on outcome selection.

*Data extraction*

Descriptive characteristics of included publications, including publication year, study details (type of study, design, number of centers involved and whether they had ethical/institutional board approval)



For peer review only - http://bmjopen.bmj.com/site/about/guidelines.xhtml

and geographical origin, were extracted. Outcome data extraction for each publication was approached systematically starting with the title. Outcomes were extracted verbatim on first mention in the publication. Data extraction included generic descriptions of outcomes (for example, safety outcomes) as well as detailed, specific descriptions (for example, specific types of adverse events). For the purpose of this review, a broad definition of an outcome was adopted to ensure that all information potentially relevant to the evaluation of innovation was captured. This included any measured or reported construct or concept relating to or occurring as a result of using the device [4]. It also included descriptions or details of events and observations that occurred during the course of the study. Outcomes that were discussed (but not necessarily measured and reported as a study endpoint) by the publication's authors as being of interest or of relevance to evaluating the device were also extracted.

For each device case study, data extraction was performed on subsequent publications until no new outcomes were seemingly being observed. The order in which publications were selected for data extraction within each case study was not specified, irrelevant of publication date or study type. Data extraction was performed by one reviewer (either NW, RM, KA, RLM, EK or ME) with double data extraction completed on 10% of papers. Any discrepancies were discussed and resolved within the study team. Data were extracted directly into an electronic purpose-designed database using REDCap (Research Electronic Data Capture) software [10].

*Data analysis: categorisation of outcomes into domains*

The complete list of verbatim outcomes extracted from all publications was reviewed by members of the study team with clinical knowledge and methodological expertise in COS development (SP, KA, RM, NW). Outcomes were categorised into a conceptual framework of outcome domains (broad classifications of aspects relating to the effects or use of the device), referring back to source publications for context if needed. The conceptual framework of outcome domains was informed by the experience of the study team and knowledge of the existing taxonomies of outcomes traditionally measured in effectiveness studies [11]. During the progression of outcome categorisation, the framework of conceptualised domains was iteratively refined, informed by the data and discussion within the study team. Domains included, for example, adverse events (that is, unfavourable signs, symptoms or incidents), clinical efficacy (for example, indications of success in treating the condition) and patient-reported outcomes. During analysis, outcomes that were considered as specific to the evaluation of innovation in the context of the included case studies (that is, were particularly relevant for considering the future use, modification or uptake/abandonment of the device) were categorised



For peer review only - http://bmjopen.bmj.com/site/about/guidelines.xhtml

as “key to evaluation of innovation”. Categorisation of outcomes into domains continued until the list of domains was comprehensive and it was possible to categorise all outcomes into the conceptualised domains. For the purpose of this review, outcomes that were categorised as “key to evaluation of innovation” were scrutinised in more detail by the study team and further categorised into sub-domains, referring to the original publication for verification of context. These sub-domains were not predefined, and categorisation was iterative and data led.

Findings were summarised using descriptive statistics, where appropriate, and narrative summaries of the outcome domains relevant to evaluating innovation.

**Patient and Public Involvement**

This review was one component of a wider study to develop a COS for surgical innovation which sought patient and public input in the concept, design, analysis and dissemination at all stages of the study[7]. This was achieved through study steering group meetings with patient and public representatives and through two NIHR Bristol Biomedical Research Centre Surgical Innovation theme patient advisory group meetings. Patients and the public were not directly involved in conducting the review or performing the analysis.



For peer review only - http://bmjopen.bmj.com/site/about/guidelines.xhtml

## Results

*Device case studies and search results*

Eleven device case studies were identified to reflect the breadth of the innovation lifecycle. Details of each device, their degree of novelty and brief descriptions of their functions are provided in Table 1.

Electronic database searches yielded 242 records, of which 114 (47%) were excluded (Figure 1). Full-text articles of the remaining 128 records were examined for eligibility. All reported on the use and/or evaluation of the device under study and were included for review (see supplementary file for references).

*Data extraction*

In total, data extraction was completed on 112/128 (87.5%) of all included publications (Figure 1). This included all publications for 10 of the 11 device case studies. The exception was the Activa Deep Brain Stimulation device. This was the last case study for which data extraction was conducted. After data extraction from 11 of the 27 included publications no new outcomes were observed to be emerging and the study team agreed that further extraction would not likely add to the conceptualisation of outcome domains. The remaining 16 publications identified for this case study were, therefore, excluded from further analysis.

*Publication characteristics*

Descriptive details of the analysed publications, presented by device case study, are displayed in Table 2. Publication dates ranged from 2009 to 2018. Most publications were from North America (n=49; 43.8%), followed by the European Economic Area (n=25; 22.3%). Publications were predominantly case series including non-comparative cohort studies (n=60; 53.6%). Other publications were single patient case reports (n=24; 21.4%), non-randomised comparative studies (n=19; 17.0%) and a small number of RCTs (n=9; 8%). Just over half of the publications reported data from a single center (n=61; 54.5%) and approximately a third reported data from multi-center studies (n=40; 35.7%). Some 11 publications (9.8%) did not report the number of centers involved. Publications reported retrospective (n=62; 55.0%) and prospective (n=50; 45.0%) data collection. A statement confirming ethical or institutional board review approval was reported in 72 (64.3%) publications.



For peer review only - http://bmjopen.bmj.com/site/about/guidelines.xhtml

For peer review only

*Categorisation of outcomes into domains*

A total of 5,926 outcomes were extracted from the 112 included publications (Table 2). The number of extracted outcomes for each device case study ranged from 117 individual outcomes (Magseed device; extracted from a total of two publications) to 1,320 outcomes (LINX reflux Management System; extracted from a total of 22 publications). The median number of outcomes extracted from each unique publication was 48, ranging from five to 135.

Outcome categorisation continued until 2689 (45.4%) outcomes had been categorised to derive a conceptual framework of five broad outcome domains. The conceptual framework included: 1) anticipated or procedural events (defined as expected or routine incidents or consequences, n=1192; 44.3%), 2) clinical, technical or common data element parameters (defined as routinely-measured data variables of interest to the clinical specialty [12], n=745; 27.7%), 3) device function (defined as technical or operational performance parameters specific to the device, n=446; 16.6%), 4) patient-reported outcomes (n= 240; 8.9%,) and; 5) outcomes key to evaluating innovation (that is, considered to be particularly relevant to the future use, modification or uptake/abandonment of the device, n=66, 2.5%) (Figure 2). The remaining 3237 (54.6%) outcomes were not categorised as the list of conceptualised domains was agreed by the study team to be comprehensive and exhaustive (that is, it was possible to categorise subsequent outcomes into the existing domains) or because outcomes were too broad in their description to make categorisation possible (for example "safety").

*Outcomes key to evaluating innovation*

Outcomes categorised as key to the evaluation of innovation (n=66; 2.5%) were further categorised into three sub-domains: surgeon/operator experience, unanticipated events, and modifications.

Some 40 (1.5%) outcomes were categorised as relating to surgeon/operator experience. These included, for example, ease of using the new device, convenience and satisfaction. For the majority of the included case studies, the operator of the device was the surgeon. In the case study of the AeroForm tissue expander, the device was operated by the patient following discharge from hospital. An example of an outcome relevant to patients' experience of operating the device included "very easy" use of the dosage controller [13].

Some 15 (0.6%) outcomes were categorised as unanticipated events. These included adverse events related to the use of the device that were reported by the authors as unexpected or not previously encountered or anticipated. An example of an outcome coded as an unanticipated event was an inability to dislodge the device in a retrieval attempt in the case of the leadless cardiac pacemaker



For peer review only - http://bmjopen.bmj.com/site/about/guidelines.xhtml

(Micra LCP) [14]. A further example included fatal intestinal injury during surgery in the case study of the Align urethral support system [15].

Some 11 (0.4%) outcomes were categorised as modifications. These included adjustments to the device, for example, redesign of the internal membrane of the AeroForm tissue expander following testing during its development [16]. Authors noted how the expander bulk was decreased to minimise the risk of underexpansion and erosion, and therefore potential harm, in subsequent patients [16]. Detail on modification outcomes, however, were rarely reported in any of the other device case studies. Of the 11 modification outcomes, seven were from the Aeroform case study with the remaining four extracted from only three of the other case studies. Modifications were often described by authors in general terms and without specific descriptions, such as “design modifications” in the example of the Accolade hip stem [17] and “modifications to the liner” or “midtrial modifications to the expander” in the example of the AeroForm device [18].

For peer review only

## Discussion

This review identified a broad range of outcomes identified, selected and reported in studies describing the introduction and evaluation of a purposive sample of novel devices, chosen to reflect the breadth of surgical innovation. Although relatively few in number, outcomes considered key to the evaluation of innovation were identified from these empirical case studies, distinct from most outcomes shared with effective studies [11] . These were broadly categorised as surgeon/operator experience, unanticipated events and modifications. Although infrequently reported in the studies included in this review, we consider these outcomes to be important drivers of innovation at the early stage of the device lifecycle and key to assessing procedures with new medical devices.

Recent systematic reviews have demonstrated that outcome reporting in studies of innovative surgical procedures and devices focus on short term, clinical and technical outcomes [19, 20]. These outcomes are critical to assess in any early phase study and are required for US Food and Drug Administration (FDA) and the UK Medicines and Healthcare products Regulatory Agency (MHRA) approval [21, 22]. These types of outcomes are also the recommended focus of early-phase studies (stages 1 and 2) in the IDEAL (Idea, Development, Exploration, Assessment, Long-term Follow-up) framework for the introduction and evaluation of new surgical procedures and devices (IDEAL-D) [23, 24]. The current review, however, has studied outcome selection and reporting in depth in empirical case studies, in order to identify innovation-specific outcomes potentially important to evaluating the process of innovation that have not explicitly been specified as key outcomes in existing guidance. Assessing surgeon/operator experience of using a new device, for example, can highlight issues and provide valuable feedback for the device developers and clinical colleagues, playing an important role in making improvements in future versions of the device or the way in which the device is inserted or used. User experience may also determine whether a device is adopted into practice or abandoned, further underlining the relevance of this outcome as a key driver to innovation. The examples of unanticipated events identified in this review are also vitally important to the introduction and evaluation of innovation as they may represent patient safety issues that should be shared with clinicians undertaking the procedure in the future to minimise patient harm. It is recognised that unanticipated events are commonly reported in later-phase effectiveness studies such as RCTs, in particular in studies evaluating pharmaceuticals. In the context of early-phase studies of surgical devices, however, they are particularly important to highlight unexpected problems requiring modifications to the device or the procedure for using or inserting it. Such modifications may improve the efficacy of the device, its ease of use or, perhaps most importantly, reduce the risk of patient



For peer review only - http://bmjopen.bmj.com/site/about/guidelines.xhtml

For peer review only

harm. Reporting unanticipated outcomes and modifications in early stages of the introduction of a device is key to shared learning relating to innovation with the wider surgical community, providing vital information to surgeons using the device in the future and to avoid repeated mistakes or harms.

This review examined a broad range of case studies of novel devices across the innovation lifecycle and across different surgical specialties. There are, however, some limitations. Case studies were identified by asking specialists known to the study team rather than a wider survey. A purposively-selected sample covering a range of specialties and stage of innovation were subsequently included. It is acknowledged that the 11 included case studies are not an exhaustive sample and may not be representative of all innovative devices. This study, however, was one of many data sources to inform the list of outcomes to consider development of the COS, which used triangulated data from published literature, qualitative interviews and regulatory body documents[7]. Identification of publications involved database searches performed using the device trade name as the key search term. It is recognised that some relevant studies conducted before the trade name was established may have been missed. It is also acknowledged that a small number of outcomes may have been missed during data extraction, with only 10% of papers undergoing a second reviewer independent check. It is unlikely, however, that this would have altered the conceptualised framework of outcome domains. During analysis, a broad approach to categorise outcomes was taken, using a conceptual framework that evolved during the iterative process of data analysis. Categorisation of outcomes during the earlier stages of analysis, therefore, may not represent how they may have been defined later in the evolution of the conceptual framework. Furthermore, each outcome was categorised only to a single outcome domain. For some outcomes, more than one domain many have been applicable, for example, "complete tumor excision" could be categorised as a procedural event or a clinical parameter. Decisions were based on reviewers' judgement which limits the reproducibility of the exact number of outcomes categorised to each domain. Outcomes categorised as key to evaluation of innovation were, however, scrutinised in more detail by referring to the contextual information in the original publication to better understand the nature of the outcome. Furthermore, the aim of this review was to identify outcomes and domains of specific relevance to innovation and the methodology used was successful in achieving this goal. Finally, this review only included case studies of novel surgical devices. These were specifically selected to identify outcomes that may have relevance to the introduction and evaluation of medical devices, hypothesising that there may be innovation outcomes specific to device use. By focusing on devices, this review may have failed to identify key innovation outcomes of relevance to the introduction of surgical techniques not involving a device. This review,



For peer review only - http://bmjopen.bmj.com/site/about/guidelines.xhtml

however, was only one of multiple data sources used to inform the list of outcomes for later COS development [7, 25, 26].

Improvements in the processes for the introduction and evaluation of surgical innovations are a recognised need. The recent UK Independent Medicines and Medical Devices Safety Review, commissioned after the catastrophic consequences and harms experienced by some patients, highlighted the gaps and inefficiencies in the current regulatory systems [27]. Our work has explored the complexities surrounding the introduction of new procedures/devices into routine practice, highlighting shortcomings and inconsistencies in how surgical innovation is evaluated and reported [19, 20, 28]. This has highlighted the need for consensus on the outcomes that are essential to measure to support the safe and efficient introduction and evaluation of new surgical procedures and devices.

Findings from the current study, combined with outcomes identified from other data sources [25, 26], later informed an international Delphi consensus process with key stakeholders to agree on a core set of outcomes for use in  all studies evaluating new surgical procedures/devices (the COHESIVE core outcome set) [8]. Mandatory measurement and reporting of this core outcome set in all future studies of new surgical procedures and devices will ensure that key outcomes of relevance to innovation that are important to patients and key stakeholder are included. The aim of the COS is to improve standardisation and consistency across studies, allowing results to be compared and combined. Furthermore, reporting and dissemination of these outcomes in real-time would inform surgeons, patients, device manufacturers and other key stakeholders of both the benefits and harms of new surgical procedures/devices in a timely manner and promote their safe, transparent and efficient evaluation and introduction and into clinical practice, avoiding patient harms. Work is currently underway to encourage the effective uptake of the new COS, to identify how to best measure these novel outcome domains, and to develop a real-time reporting and sharing platform to streamline the process of surgical innovation to support surgeon-innovators and improve patient outcomes.



For peer review only - http://bmjopen.bmj.com/site/about/guidelines.xhtml

## Footnotes

**Author contributions:** NW and RCM participated in the research design, performance of the research, data analysis and writing of the paper; CH participated in the performance of the research and data analysis; ME, RLM, EK participated in the performance of the research; NS, AM and BG participated in the intellectual content, research design and performance of the research; JM participated in the intellectual content, research design and writing of the paper, KA and SP participated in the intellectual content, research design, the performance of the research, data analysis and writing of the paper.

**Funding:** This study was funded by the National Institute for Health Research (NIHR) Biomedical Research Centre (BRC) at University Hospitals Bristol and Weston NHS Foundation Trust and the University of Bristol (BRC-1215-20011). This work was supported by the Royal College of Surgeons of England Bristol Surgical Trials Centre, the MRC ConDuCT-II (Collaboration and innovation for Difficult and Complex randomised controlled Trials In Invasive procedures) Hub for Trials Methodology Research (MR/K025643/1) and the MRC-NIHR Trials Methodology Research Partnership (TMRP). SP and AM are NIHR Clinician Scientists (NIHR CS-2016-16-019, NIHR CS-2017-17-010). NB is an MRC Clinician Scientist. BGM is a NIHR Clinical Lecturer. JB is an NIHR Senior Investigator. The views and opinions expressed in this publication are those of the authors and not necessarily those of the National Institute for Health Research, the Department of Health and Social Care, the Royal College of Surgeons of England or the Medical Research Council. The authors declare no conflicts of interest.

**Competing interests:** None declared.



For peer review only - http://bmjopen.bmj.com/site/about/guidelines.xhtml

## References


1. Cohen, D., *How safe are metal-on-metal hip implants?* British Medical Journal, 2012. **344**: p. e1410.
2. Heneghan, C., et al., *Transvaginal mesh failure: lessons for regulation of implantable devices.* British Medical Journal, 2017. **359**: p. j5515.
3. Ergina, P.L., et al., *Surgical Innovation and Evaluation 2 Challenges in evaluating surgical innovation.* Lancet, 2009. **374**(9695): p. 1097-1104.
4. Gorst, S.L., et al., *Choosing Important Health Outcomes for Comparative Effectiveness Research: An Updated Review and Identification of Gaps.* PLOS ONE, 2016. **11**(12): p. e0168403.
5. Williamson, P.R., et al., *The COMET Handbook: version 1.0.* Trials, 2017. **18**(Suppl 3): p. 280.
6. Tunis, S.R., et al., *Improving the relevance and consistency of outcomes in comparative effectiveness research.* J Comp Eff Res, 2016. **5**(2): p. 193-205.
7. Avery, K., et al., *Development of reporting guidance and core outcome sets for seamless, standardised evaluation of innovative surgical procedures and devices: a study protocol for content generation and a Delphi consensus process (COHESIVE study).* BMJ Open, 2019. **9**(9): p. e029574.
8. Avery, K., et al., *A core outcome set for seamless, standardised evaluation and reporting of outcomes throughout the surgical innovation lifecycle: the COHESIVE study.* British Journal of Surgery, 2020. **107**(S4): p. 11-37.
9. US Food and Drug Administration. *Premarket Notification 510(k).* Accessed on 07/12/2020]; Available from: https://www.fda.gov/medical-devices/premarket-submissions/premarket-notification-510k.
10. Harris, P.A., et al., *Research electronic data capture (REDCap) - A metadata-driven methodology and workflow process for providing translational research informatics support.* Journal of Biomedical Informatics, 2009. **42**(2): p. 377-81.
11. Dodd, S., et al., *A taxonomy has been developed for outcomes in medical research to help improve knowledge discovery.* J Clin Epidemiol, 2018. **96**: p. 84-92.
12. Karaa, A., et al., *Common data elements for clinical research in mitochondrial disease: a National Institute for Neurological Disorders and Stroke project.* J Inherit Metab Dis, 2017. **40**(3): p. 403-414.
13. Connell, T.F., *Patient-activated controlled expansion for breast reconstruction using controlled carbon dioxide inflation: confirmation of a feasibility study.* Plast Reconstr Surg, 2014. **134**(4): p. 503e-11e.
14. Grubman, E., et al., *To retrieve, or not to retrieve: System revisions with the Micra transcatheter pacemaker.* Heart Rhythm, 2017. **14**(12): p. 1801-1806.
15. Kascak, P. and B. Kopcan, *Fatal Injury of the Small Intestine during Retropubic Sling Placement.* Case Rep Obstet Gynecol, 2015. **2015**: p. 164545.
16. Zeidler, K.R., et al., *AeroForm patient controlled tissue expansion and saline tissue expansion for breast reconstruction: a randomized controlled trial.* Ann Plast Surg, 2014. **72 Suppl 1**: p. S51-5.
17. Nam, D., et al., *An evaluation of proximal femur bone density in young, active patients undergoing total hip arthroplasty at one year postoperatively.* Hip Int, 2019. **29**(1): p. 51-57.
18. Ascherman, J.A., et al., *Carbon Dioxide-Based versus Saline Tissue Expansion for Breast Reconstruction: Results of the XPAND Prospective, Randomized Clinical Trial.* Plast Reconstr Surg, 2016. **138**(6): p. 1161-1170.
19. Pathak, S., et al., *A Systematic Review of Minimally Invasive Trans-thoracic Liver Resection to Examine Intervention Description, Governance, and Outcome Reporting of an Innovative Technique.* Ann Surg, 2020.


For peer review only



For peer review only - http://bmjopen.bmj.com/site/about/guidelines.xhtml

20. Kirkham, E.N., et al., *Systematic review of the introduction and evaluation of magnetic augmentation of the lower oesophageal sphincter for gastro-oesophageal reflux disease.* Br J Surg, 2020. **107**(1): p. 44-55.
21. US Food and Drug Administration. *Device Advice. Comprehensive Regulatory Assistance*. 28/01/2021]; Available from: https://www.fda.gov/medical-devices/device-advice-comprehensive-regulatory-assistance.
22. Medicines and Healthcare products Regulatory Agency. *Guidance - Medical devices: how to comply with the legal requirements* 2014 28/01/2021]; Available from: https://www.gov.uk/guidance/medical-devices-how-to-comply-with-the-legal-requirements.
23. McCulloch, P., et al., *No surgical innovation without evaluation: the IDEAL recommendations.* Lancet, 2009. **374**(9695): p. 1105-12.
24. Sedrakyan, A., et al., *IDEAL-D: a rational framework for evaluating and regulating the use of medical devices.* Bmj, 2016. **353**: p. i2372.
25. Hoffmann, C., et al., *A systematic review and in-depth analysis of outcome reporting in early phase studies of colorectal cancer surgical innovation.* Colorectal Dis, 2020.
26. Macefield, R.C., et al., *Outcome selection, measurement and reporting for new surgical procedures and devices: a systematic review of IDEAL/IDEAL-D studies to inform development of a core outcome set.* BJS Open, 2020.
27. Cumberlege, J. *First Do No Harm: The report of the Independent Medicines and Medical Devices Safety Review*. 2020.
28. Zahra, J., et al., *Discussing surgical innovation with patients: a qualitative study of surgeons' and governance representatives' views.* BMJ Open, 2020. **10**(11): p. e035251.

For peer review only



For peer review only - http://bmjopen.bmj.com/site/about/guidelines.xhtml

**Figure 1.** Identification and selection of included publications

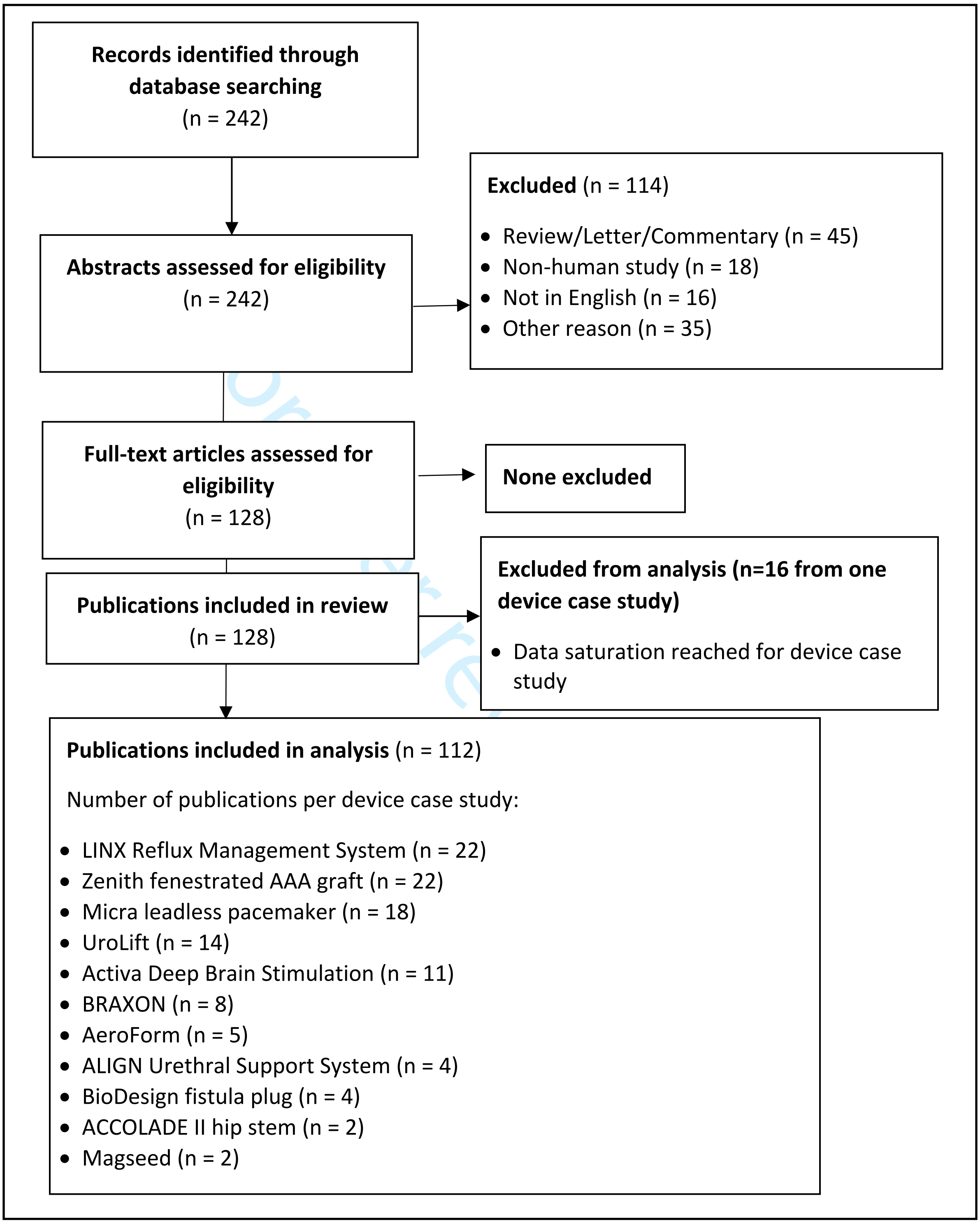




For peer review only - http://bmjopen.bmj.com/site/about/guidelines.xhtml

**Figure 2**. Categorisation of outcomes extracted from all publications (n=2689)

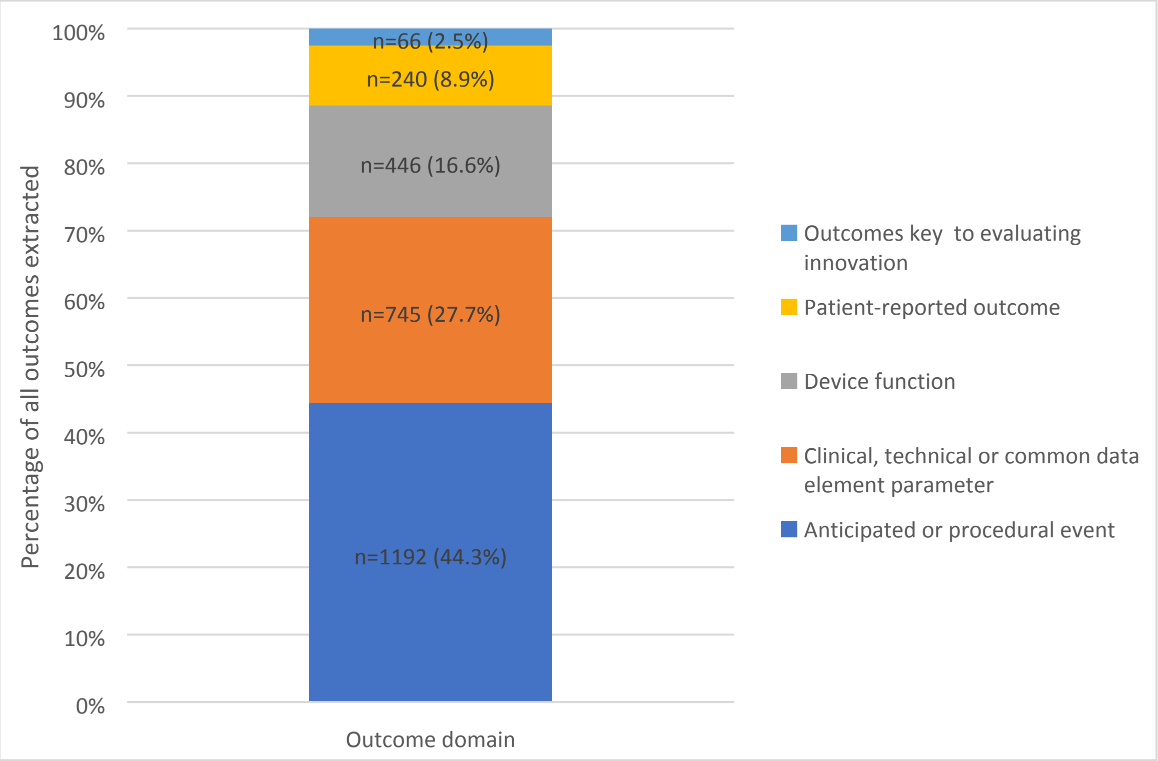


review only



For peer review only - http://bmjopen.bmj.com/site/about/guidelines.xhtml

**Table 1.** Description of the 11 medical devices selected to explore outcome selection and reporting.

| Degree of novelty* | Device tradename | Licenced by | Surgical Specialty | Description of device |
|---|---|---|---|---|
| Wholly innovative | Activa Deep Brain Stimulation | Medtronic | Neurosurgery | A dual-channel, rechargeable neurostimulator for treatment of neurological conditions including Parkinson's disease, dystonia and essential tremor. The device is typically implanted in the chest or abdomen, connected to an extension and leads, which are implanted in the brain |
| | Magseed | Endomag | Breast | A ferromagnetic localisation seed used to localise impalpable breast lesions prior to excision. |
| | UroLift | Neotract Inc | Urology | A minimally invasive treatment of benign prostatic hyperplasia or an enlarged prostate. |
| Partially innovative | AeroForm | AirXpanders | Plastic/Breast | A remote-control tissue expander filled from an internal source with carbon dioxide eliminating the need for needle injections to fill the expander. This expander makes breast reconstruction, faster and more comfortable while providing women with some degree of control over the expansion. |
| | Micra leadless pacemaker | Medtronic | Cardiology | A pacemaker implanted directly into the patient's heart. |
| | Zenith fenestrated AAA graft | Cook Medical | Vascular | An endovascular graft indicated for the endovascular treatment of patients with abdominal aortic or aorto-iliac aneurysms of a morphology not suitable for standard endovascular repair. |
| Reinvented | BRAXON | Raise Healthcare | Plastic/Breast | A pre-shaped porcine acellular dermal matrix designed to completely enclose the breast implant prior to fixation on top of the intact pectoralis major muscle for breast reconstruction. |
| | LINX Reflux Management System | Ethicon (formally Torax Medical) | Gastrointestinal | An anti-reflux device that can be inserted laparoscopically around the lower end of the esophagus for the treatment of gastroesophageal reflux disease. |
| Predicate | ACCOLADE II hip stem | Stryker | Orthopaedics | A morphometric wedge femoral hip stem, merging conventional tapered wedge femoral stem design with size specific medial curvature to more closely fit a broad range of bone sizes and shapes of today's patient population. |
| | ALIGN Urethral Support System | Bard Medical | Urology/ Gynaecology | A macroporous lightweight polypropylene mesh designed to treat cases of stress urinary incontinence as well as pelvic organ prolapse |
| | BioDesign fistula plug | Cook Medical | Gastrointestinal | A fistula plug for implantation to reinforce soft tissue for the repair of recto-vaginal or anorectal fistulas. |

For peer review only

*Wholly innovative: a device that represents a completely new approach to solving a clinical problem; Partially innovative: a device that is broadly similar in function to one already in use but differs in at least one significant way; Reinvented: a modification of a device or technique that was previously abandoned for complications; Predicate: a device introduced via the FDA predicate 510(k) pathway on the basis of equivalence

For peer review only

**Table 2**. Publication characteristics and number of outcomes extracted for the 11 case studies of innovative medical devices.

| Degree of novelty* | | Wholly innovative | | | Partially innovative | | | Reinvented | | Predicate | | |
|---|---|---|---|---|---|---|---|---|---|---|---|---|
| | **Overall** | Activa Deep Brain Stimulation | Magseed | UroLift | AeroForm | Micra leadless pacemaker | Zenith fenestrated AAA graft | BRAXON | LINX Reflux Management System | ACCOLADE II hip stem | ALIGN Urethral Support System | BioDesign fistula plug |
| Number of publications included | **112** | 11 | 2 | 14 | 5 | 18 | 22 | 8 | 22 | 2 | 4 | 4 |
| Year of publication (range) | **2009-2018** | 2017-2018 | 2018 | 2013-2018 | 2014-2017 | 2015-2018 | 2016-2018 | 2016-2018 | 2010-2018 | 2014-2018 | 2013-2015 | 2009-2015 |
| Country of origin, n (%) | | | | | | | | | | | | |
| EEA | **25 (22.3)** | 3 | 0 | 2 | 0 | 3 | 4 | 4 | 5 | 0 | 2 | 2 |
| North America | **49 (43.8)** | 6 | 1 | 3 | 3 | 2 | 15 | 0 | 15 | 2 | 2 | 0 |
| UK | **12 (10.7)** | 1 | 1 | 3 | 0 | 1 | 2 | 3 | 0 | 0 | 0 | 1 |
| Multiple | **17 (15.2)** | 0 | 0 | 5 | 0 | 10 | 0 | 0 | 2 | 0 | 0 | 0 |
| Other | **9 (8.0)** | 1 | 0 | 1 | 2 | 2 | 1 | 1 | 0 | 0 | 0 | 1 |
| Type of publication, n (%) | | | | | | | | | | | | |
| Case report | **24 (21.4)** | 3 | 0 | 2 | 0 | 7 | 3 | 1 | 7 | 0 | 1 | 0 |
| Case series | **60 (53.6)** | 6 | 2 | 6 | 1 | 2 | 17 | 7 | 11 | 2 | 2 | 4 |
| Non-randomised comparative study | **19 (17.0)** | 2 | 0 | 1 | 2 | 9 | 2 | 0 | 3 | 0 | 0 | 0 |

For peer review only

| | | | | | | | | | | | | |
|---|---|---|---|---|---|---|---|---|---|---|---|---|
| Randomised controlled trial | **9 (8.0)** | 0 | 0 | 5 | 2 | 0 | 0 | 0 | 1 | 0 | 1 | 0 |
| Multi/single center, n (%) | | | | | | | | | | | | |
| Single center | **61 (54.5)** | 8 | 1 | 5 | 3 | 7 | 15 | 5 | 10 | 1 | 4 | 2 |
| Multicenter | **40 (35.7)** | 2 | 1 | 6 | 2 | 11 | 5 | 2 | 9 | 0 | 0 | 2 |
| Not reported | **11 (9.8)** | 1 | 0 | 3 | 0 | 0 | 2 | 1 | 3 | 1 | 0 | 0 |
| Data collection, n (%) | | | | | | | | | | | | |
| Retrospective | **62 (55)** | 6 | 0 | 8 | 0 | 8 | 15 | 4 | 15 | 0 | 3 | 3 |
| Prospective | **50 (45)** | 5 | 2 | 6 | 5 | 10 | 7 | 4 | 7 | 2 | 1 | 1 |
| Ethical /Institutional review board approved, n (%) | | | | | | | | | | | | |
| Yes | **72 (64.3)** | 7 | 2 | 7 | 3 | 10 | 14 | 4 | 17 | 2 | 3 | 3 |
| No | **40 (35.7)** | 4 | 0 | 7 | 2 | 8 | 8 | 4 | 5 | 0 | 1 | 1 |
| Number of outcomes extracted | **5,926** | 635 | 117 | 423 | 264 | 1,012 | 1,070 | 611 | 1,320 | 122 | 233 | 119 |

*Wholly innovative: a device that represents a completely new approach to solving a clinical problem; Partially innovative: a device that is broadly similar in function to one already in use but differs in at least one significant way; Reinvented: a modification of a device or technique that was previously abandoned for complications; Predicate: a device introduced via the FDA predicate 510(k) pathway on the basis of equivalence

For peer review only

Publications included in the review (n=128)

1. Afzal, M.R., et al., *Techniques for successful early retrieval of the Micra transcatheter pacing system: A worldwide experience.* Heart Rhythm, 2018. **15**(6): p. 841-846.
2. Ahmed, F.Z., et al., *Totally Leadless Dual-Device Implantation for Combined Spontaneous Ventricular Tachycardia Defibrillation and Pacemaker Function: A First Report.* Can J Cardiol, 2017. **33**(8): p. 1066.e5-1066.e7.
3. Alicuben, E.T., et al., *Worldwide Experience with Erosion of the Magnetic Sphincter Augmentation Device.* J Gastrointest Surg, 2018. **22**(8): p. 1442-1447.
4. Ascherman, J.A., et al., *Carbon Dioxide-Based versus Saline Tissue Expansion for Breast Reconstruction: Results of the XPAND Prospective, Randomized Clinical Trial.* Plast Reconstr Surg, 2016. **138**(6): p. 1161-1170.
5. Asti, E., et al., *Longitudinal comparison of quality of life in patients undergoing laparoscopic Toupet fundoplication versus magnetic sphincter augmentation: Observational cohort study with propensity score analysis.* Medicine (Baltimore), 2016. **95**(30): p. e4366.
6. Asti, E., et al., *Removal of the Magnetic Sphincter Augmentation Device: Surgical Technique and Results of a Single-center Cohort Study.* Ann Surg, 2017. **265**(5): p. 941-945.
7. Bardoli, A.D., W.S.J. Taylor, and W. Mahmalji, *Can the UroLift prostatic implant device treat the symptoms of benign prostatic hypertrophy, avoid sexual dysfunction and reduce hospital TURP waiting times? A single centre, single surgeon experience and review of the literature.* Aging Male, 2017. **20**(3): p. 192-197.
8. Bauer, M., et al., *Endoluminal perforation of a magnetic antireflux device.* Surg Endosc, 2015. **29**(12): p. 3806-10.
9. Bell, R., et al., *Laparoscopic magnetic sphincter augmentation versus double-dose proton pump inhibitors for management of moderate-to-severe regurgitation in GERD: a randomized controlled trial.* Gastrointest Endosc, 2019. **89**(1): p. 14-22.e1.
10. Berna, G., et al., *Evaluation of a novel breast reconstruction technique using the Braxon(®) acellular dermal matrix: a new muscle-sparing breast reconstruction.* ANZ J Surg, 2017. **87**(6): p. 493-498.
11. Biswal, N.C., et al., *UroLift as a surrogate for fiducial markers in IGRT planning of prostate cancer in BPH patients.* Pract Radiat Oncol, 2018. **8**(4): p. e231-e233.
12. Blom, J., et al., *Results of collagen plug occlusion of anal fistula: a multicentre study of 126 patients.* Colorectal Dis, 2014. **16**(8): p. 626-30.
13. Blumenfeld, Z., et al., *Sixty-hertz stimulation improves bradykinesia and amplifies subthalamic low-frequency oscillations.* Mov Disord, 2017. **32**(1): p. 80-88.
14. Bonavina, L., et al., *Laparoscopic sphincter augmentation device eliminates reflux symptoms and normalizes esophageal acid exposure: one- and 2-year results of a feasibility trial.* Ann Surg, 2010. **252**(5): p. 857-62.
15. Bozkurt, A., et al., *Prostatic Urethral Lift: A New Minimally Invasive Treatment for Lower Urinary Tract Symptoms Secondary to Benign Prostatic Hyperplasia.* Urol Int, 2016. **96**(2): p. 202-6.
16. Buckley, F.P., 3rd, et al., *Favorable results from a prospective evaluation of 200 patients with large hiatal hernias undergoing LINX magnetic sphincter augmentation.* Surg Endosc, 2018. **32**(4): p. 1762-1768.
17. Cantwell, A.L., et al., *Multicentre prospective crossover study of the 'prostatic urethral lift' for the treatment of lower urinary tract symptoms secondary to benign prostatic hyperplasia.* BJU Int, 2014. **113**(4): p. 615-22.
18. Chelvarajah, R., et al., *Shielded battery syndrome: a new hardware complication of deep brain stimulation.* Stereotact Funct Neurosurg, 2012. **90**(2): p. 113-7.
19. Chin, P. and P. Robertson, *Medium-term efficacy of the prostatic urethral lift.* Transl Androl Urol, 2017. **6**(Suppl 2): p. S122-s132.

For peer review only

20. Chinitz, L., et al., *Accelerometer-based atrioventricular synchronous pacing with a ventricular leadless pacemaker: Results from the Micra atrioventricular feasibility studies.* Heart Rhythm, 2018. **15**(9): p. 1363-1371.
21. Connell, T.F., *Patient-activated controlled expansion for breast reconstruction using controlled carbon dioxide inflation: confirmation of a feasibility study.* Plast Reconstr Surg, 2014. **134**(4): p. 503e-11e.
22. Connell, T.F., *Results from the ASPIRE study for breast reconstruction utilizing the AeroForm™ patient controlled carbon dioxide-inflated tissue expanders.* J Plast Reconstr Aesthet Surg, 2015. **68**(9): p. 1255-61.
23. de Souza, L.R., et al., *Editor's Choice - Comparison of Renal Outcomes in Patients Treated by Zenith(®) Fenestrated and Zenith(®) Abdominal Aortic Aneurysm Stent grafts in US Prospective Pivotal Trials.* Eur J Vasc Endovasc Surg, 2017. **53**(5): p. 648-655.
24. Desart, K., et al., *Gastroesophageal Reflux Management with the LINX® System for Gastroesophageal Reflux Disease Following Laparoscopic Sleeve Gastrectomy.* J Gastrointest Surg, 2015. **19**(10): p. 1782-6.
25. Dorsey, C., V. Chandra, and J.T. Lee, *The "terrace technique"--totally endovascular repair of a type IV thoracoabdominal aortic aneurysm.* Ann Vasc Surg, 2014. **28**(6): p. 1563.e11-6.
26. Duray, G.Z., et al., *Long-term performance of a transcatheter pacing system: 12-Month results from the Micra Transcatheter Pacing Study.* Heart Rhythm, 2017. **14**(5): p. 702-709.
27. El-Chami, M., et al., *Impact of operator experience and training strategy on procedural outcomes with leadless pacing: Insights from the Micra Transcatheter Pacing Study.* Pacing Clin Electrophysiol, 2017. **40**(7): p. 834-842.
28. England, A., et al., *Migration of fenestrated aortic stent grafts.* J Vasc Surg, 2013. **57**(6): p. 1543-52.
29. England, A., M. García-Fiñana, and R.G. McWilliams, *Multicenter retrospective investigation into migration of fenestrated aortic stent grafts.* J Vasc Surg, 2015. **62**(4): p. 884-92.
30. Farber, M.A., R. Vallabhaneni, and W.A. Marston, *"Off-the-shelf" devices for complex aortic aneurysm repair.* J Vasc Surg, 2014. **60**(3): p. 579-84.
31. Filgate, R., A. Thomas, and M. Ballal, *Treatment of foregut fistula with biologic plugs.* Surg Endosc, 2015. **29**(7): p. 2006-12.
32. Fisher, B., et al., *Battery Longevity Comparison of Two Commonly Available Dual Channel Implantable Pulse Generators Used for Subthalamic Nucleus Stimulation in Parkinson's Disease.* Stereotact Funct Neurosurg, 2018. **96**(3): p. 151-156.
33. Gallitto, E., et al., *Results of standard suprarenal fixation endografts for abdominal aortic aneurysms with neck length ≤10 mm in high-risk patients unfit for open repair and fenestrated endograft.* J Vasc Surg, 2016. **64**(3): p. 563-570.e1.
34. Gallitto, E., et al., *Endovascular Repair of Thoracoabdominal Aortic Aneurysm in High-Surgical Risk Patients: Fenestrated and Branched Endografts.* Ann Vasc Surg, 2017. **40**: p. 170-177.
35. Gerdes, C. and J.C. Nielsen, *Retrieval of Medtronic Micra Transcatheter Pacing System after tether removal.* Europace, 2016. **18**(8): p. 1202.
36. Gonsalves, S., et al., *Assessment of the efficacy of the rectovaginal button fistula plug for the treatment of ileal pouch-vaginal and rectovaginal fistulas.* Dis Colon Rectum, 2009. **52**(11): p. 1877-81.
37. Gratzke, C., et al., *Prostatic urethral lift vs transurethral resection of the prostate: 2-year results of the BPH6 prospective, multicentre, randomized study.* BJU Int, 2017. **119**(5): p. 767-775.
38. Greenberg, R.K., et al., *Intermediate results of a United States multicenter trial of fenestrated endograft repair for juxtarenal abdominal aortic aneurysms.* J Vasc Surg, 2009. **50**(4): p. 730-737.e1.

For peer review only

39. Grubman, E., et al., *To retrieve, or not to retrieve: System revisions with the Micra transcatheter pacemaker.* Heart Rhythm, 2017. **14**(12): p. 1801-1806.
40. Halak, M., M.A. Goodman, and S.R. Baker, *The fate of target visceral vessels after fenestrated endovascular aortic repair--general considerations and mid-term results.* Eur J Vasc Endovasc Surg, 2006. **32**(2): p. 124-8.
41. Harvey, J.R., et al., *Safety and feasibility of breast lesion localization using magnetic seeds (Magseed): a multi-centre, open-label cohort study.* Breast Cancer Res Treat, 2018. **169**(3): p. 531-536.
42. Hawasli, A., A. Phillips, and M. Tarboush, *Laparoscopic management of reflux after Roux-en-Y gastric bypass using the LINX system and repair of hiatal hernia: a case report.* Surg Obes Relat Dis, 2016. **12**(5): p. e51-e54.
43. Hawasli, A., M. Tarakji, and M. Tarboush, *Laparoscopic management of severe reflux after sleeve gastrectomy using the LINX(®) system: Technique and one year follow up case report.* Int J Surg Case Rep, 2017. **30**: p. 148-151.
44. Helmers, A.K., et al., *Comparison of the Battery Life of Nonrechargeable Generators for Deep Brain Stimulation.* Neuromodulation, 2018. **21**(6): p. 593-596.
45. Hemm, S., et al., *Contact position analysis of deep brain stimulation electrodes on post-operative CT images.* Acta Neurochir (Wien), 2009. **151**(7): p. 823-9; discussion 829.
46. Henderson, F., Jr. and I. Takacs, *Rapid and Minimally Traumatic Replacement of Stimulator Extension Cables: Technical Note on a Novel Use for Sternal Wire.* World Neurosurg, 2017. **97**: p. 547-550.
47. Herron, J.A., et al., *Chronic electrocorticography for sensing movement intention and closed-loop deep brain stimulation with wearable sensors in an essential tremor patient.* J Neurosurg, 2017. **127**(3): p. 580-587.
48. Holm, N., A. Müller, and R. Zbinden, *Complications with the MICRA TPS Pacemaker System: Persistent Complete Heart Block and Late Capture Failure.* Pacing Clin Electrophysiol, 2017. **40**(4): p. 455-456.
49. Iqbal, F.M., A. Bhatnagar, and R. Vidya, *Host Integration of an Acellular Dermal Matrix: Braxon Mesh in Breast Reconstruction.* Clin Breast Cancer, 2016. **16**(6): p. e209-e211.
50. Iqbal, M., et al., *Low power HOLEP after failed urolift: A case report using 50 Watt laser.* Urol Case Rep, 2018. **16**: p. 114-115.
51. Issa, K., et al., *Radiographic fit and fill analysis of a new second-generation proximally coated cementless stem compared to its predicate design.* J Arthroplasty, 2014. **29**(1): p. 192-8.
52. Jafferbhoy, S., et al., *Early multicentre experience of pre-pectoral implant based immediate breast reconstruction using Braxon(®).* Gland Surg, 2017. **6**(6): p. 682-688.
53. Kalder, J., et al., *Anatomic changes of target vessels after fenestrated and branched aortic aneurysm repair.* J Cardiovasc Surg (Torino), 2014. **55**(2 Suppl 1): p. 115-21.
54. Kaminska, M., et al., *Rechargeable deep brain stimulators in the management of paediatric dystonia: well tolerated with a low complication rate.* Stereotact Funct Neurosurg, 2012. **90**(4): p. 233-9.
55. Kaminska, M., et al., *Complications of Deep Brain Stimulation (DBS) for dystonia in children - The challenges and 10 year experience in a large paediatric cohort.* Eur J Paediatr Neurol, 2017. **21**(1): p. 168-175.
56. Kascak, P. and B. Kopcan, *Fatal Injury of the Small Intestine during Retropubic Sling Placement.* Case Rep Obstet Gynecol, 2015. **2015**: p. 164545.
57. Kawasaki, A., et al., *Comparing the risk of urethrolysis for the treatment of voiding dysfunction between two retropubic mesh slings: a case-control study.* Int Urogynecol J, 2013. **24**(4): p. 589-94.
58. Keehn, A., et al., *UroLift in Place of Fiducial Markers for Patients With Benign Prostatic Hyperplasia Undergoing External Beam Radiation Therapy.* Urology, 2017. **104**: p. 230-234.

For peer review only

59. Kelley, K. and J. Kim, *Human Factors Validation of the AeroForm Tissue Expander System for Breast Reconstruction.* Plast Surg Nurs, 2017. **37**(3): p. 100-102.
60. Kerwin, S.A., M.J. Mayotte, and C.C. Gornick, *Transcatheter pacemaker implantation in a patient with a bioprosthetic tricuspid valve.* J Interv Card Electrophysiol, 2015. **44**(1): p. 89-90.
61. Kitagawa, A., et al., *Zenith p-branch standard fenestrated endovascular graft for juxtarenal abdominal aortic aneurysms.* J Vasc Surg, 2013. **58**(2): p. 291-300.
62. Kypta, A., et al., *Dawn of a new era: the completely interventionally treated patient.* BMJ Case Rep, 2016. **2016**.
63. Lala, S., et al., *Superior mesenteric artery outcomes after fenestrated endovascular aortic aneurysm repair.* J Vasc Surg, 2016. **64**(3): p. 692-7.
64. Lin, J.-P., et al., *Bilateral globus pallidus internus deep brain stimulation for dyskinetic cerebral palsy supports success of cochlear implantation in a 5-year old ex-24 week preterm twin with absent cerebellar hemispheres.* European Journal of Paediatric Neurology, 2017. **21**(1): p. 202-213.
65. Lipham, J.C., et al., *The LINX® reflux management system: confirmed safety and efficacy now at 4 years.* Surg Endosc, 2012. **26**(10): p. 2944-9.
66. Lloyd, M., et al., *Rate adaptive pacing in an intracardiac pacemaker.* Heart Rhythm, 2017. **14**(2): p. 200-205.
67. Louie, B.E., et al., *Objective Evidence of Reflux Control After Magnetic Sphincter Augmentation: One Year Results From a Post Approval Study.* Ann Surg, 2019. **270**(2): p. 302-308.
68. Lyon, J.W., et al., *First experience with the use of a collagen fistula plug to treat enterocutaneous fistulas.* J Vasc Interv Radiol, 2013. **24**(10): p. 1559-65.
69. Madsen, A.M., et al., *A cohort study comparing a single-incision sling with a retropubic midurethral sling.* Int Urogynecol J, 2014. **25**(3): p. 351-8.
70. Maling, N., et al., *Biophysical basis of subthalamic local field potentials recorded from deep brain stimulation electrodes.* J Neurophysiol, 2018. **120**(4): p. 1932-1944.
71. Maruccia, M., G. Di Taranto, and M.G. Onesti, *One-stage muscle-sparing breast reconstruction in elderly patients: A new tool for retaining excellent quality of life.* Breast J, 2018. **24**(2): p. 180-183.
72. Maruccia, M., et al., *One-stage breast reconstruction techniques in elderly patients to preserve quality of life.* Eur Rev Med Pharmacol Sci, 2016. **20**(24): p. 5058-5066.
73. McAdams, S., et al., *Holmium Laser Enucleation of the Prostate After Prostatic Urethral Lift Surgery: Feasibility and Technical Considerations from a Multi-Institutional Case Series.* J Endourol, 2017. **31**(8): p. 774-779.
74. McNicholas, T.A., et al., *Minimally invasive prostatic urethral lift: surgical technique and multinational experience.* Eur Urol, 2013. **64**(2): p. 292-9.
75. Morita, J., et al., *Retrieval of a Micra transcatheter pacing system in a heart with a preexisting lead.* Indian Pacing Electrophysiol J, 2018. **18**(5): p. 183-184.
76. Nam, D., et al., *An evaluation of proximal femur bone density in young, active patients undergoing total hip arthroplasty at one year postoperatively.* Hip international : the journal of clinical and experimental research on hip pathology and therapy, 2019. **29**(1): p. 51-57.
77. Neumann, W.J., et al., *Long term correlation of subthalamic beta band activity with motor impairment in patients with Parkinson's disease.* Clin Neurophysiol, 2017. **128**(11): p. 2286-2291.
78. Niemann, M., et al., *Longevity of Implantable Pulse Generators in Bilateral Deep Brain Stimulation for Movement Disorders.* Neuromodulation, 2018. **21**(6): p. 597-603.
79. Ondo, W.G., C. Meilak, and K.D. Vuong, *Predictors of battery life for the Activa Soletra 7426 Neurostimulator.* Parkinsonism Relat Disord, 2007. **13**(4): p. 240-2.

For peer review only

80. Onesti, M.G., et al., *Clinical, histological, and ultrasound follow-up of breast reconstruction with one-stage muscle-sparing "wrap" technique: A single-center experience.* J Plast Reconstr Aesthet Surg, 2017. **70**(11): p. 1527-1536.
81. O'Rawe, J.A., et al., *Integrating precision medicine in the study and clinical treatment of a severely mentally ill person.* PeerJ, 2013. **1**: p. e177.
82. Parmar, A.D., et al., *Two-Stage Explantation of a Magnetic Lower Esophageal Sphincter Augmentation Device Due to Esophageal Erosion.* J Laparoendosc Adv Surg Tech A, 2017. **27**(8): p. 829-833.
83. Perez, J., et al., *Rechargeable or Nonrechargeable Deep Brain Stimulation in Dystonia: A Cost Analysis.* Neuromodulation, 2017. **20**(3): p. 243-247.
84. Piccini, J.P., et al., *Long-term outcomes in leadless Micra transcatheter pacemakers with elevated thresholds at implantation: Results from the Micra Transcatheter Pacing System Global Clinical Trial.* Heart Rhythm, 2017. **14**(5): p. 685-691.
85. Pollo, C., et al., *Magnetic resonance artifact induced by the electrode Activa 3389: an in vitro and in vivo study.* Acta Neurochir (Wien), 2004. **146**(2): p. 161-4.
86. Price, E.R., et al., *Initial Clinical Experience With an Inducible Magnetic Seed System for Preoperative Breast Lesion Localization.* AJR Am J Roentgenol, 2018. **210**(4): p. 913-917.
87. Quinn, E.J., et al., *Beta oscillations in freely moving Parkinson's subjects are attenuated during deep brain stimulation.* Mov Disord, 2015. **30**(13): p. 1750-8.
88. Ramirez de Noriega, F., et al., *Constant Current versus Constant Voltage Subthalamic Nucleus Deep Brain Stimulation in Parkinson's Disease.* Stereotact Funct Neurosurg, 2015. **93**(2): p. 114-121.
89. Reynolds, D., et al., *A Leadless Intracardiac Transcatheter Pacing System.* N Engl J Med, 2016. **374**(6): p. 533-41.
90. Reynolds, J.L., et al., *Magnetic sphincter augmentation with the LINX device for gastroesophageal reflux disease after U.S. Food and Drug Administration approval.* Am Surg, 2014. **80**(10): p. 1034-8.
91. Reynolds, J.L., et al., *Laparoscopic Magnetic Sphincter Augmentation vs Laparoscopic Nissen Fundoplication: A Matched-Pair Analysis of 100 Patients.* J Am Coll Surg, 2015. **221**(1): p. 123-8.
92. Ritter, P., et al., *Early performance of a miniaturized leadless cardiac pacemaker: the Micra Transcatheter Pacing Study.* Eur Heart J, 2015. **36**(37): p. 2510-9.
93. Roberts, P.R., et al., *A leadless pacemaker in the real-world setting: The Micra Transcatheter Pacing System Post-Approval Registry.* Heart Rhythm, 2017. **14**(9): p. 1375-1379.
94. Roehrborn, C.G., et al., *Five year results of the prospective randomized controlled prostatic urethral L.I.F.T. study.* Can J Urol, 2017. **24**(3): p. 8802-8813.
95. Roehrborn, C.G., et al., *Three year results of the prostatic urethral L.I.F.T. study.* Can J Urol, 2015. **22**(3): p. 7772-82.
96. Rona, K.A., et al., *Efficacy of magnetic sphincter augmentation in patients with large hiatal hernias.* Surg Endosc, 2017. **31**(5): p. 2096-2102.
97. Rona, K.A., et al., *Hiatal hernia recurrence following magnetic sphincter augmentation and posterior cruroplasty: intermediate-term outcomes.* Surg Endosc, 2018. **32**(7): p. 3374-3379.
98. Rukstalis, D., et al., *24-month durability after crossover to the prostatic urethral lift from randomised, blinded sham.* BJU Int, 2016. **118 Suppl 3**: p. 14-22.
99. Salvador, R., et al., *Esophageal Penetration of the Magnetic Sphincter Augmentation Device: History Repeats Itself.* J Laparoendosc Adv Surg Tech A, 2017. **27**(8): p. 834-838.
100. Sammartino, F., et al., *3-Tesla MRI in patients with fully implanted deep brain stimulation devices: a preliminary study in 10 patients.* J Neurosurg, 2017. **127**(4): p. 892-898.
101. Shahverdyan, R., et al., *Technical feasibility of endovascular aortoiliac aneurysm repair combining Anaconda fenestrated and Zenith iliac side-branched stent grafts.* J Vasc Surg, 2015. **61**(5): p. 1324-8.

For peer review only

102. Shahverdyan, R., et al., *F-EVAR does not Impair Renal Function more than Open Surgery for Juxtarenal Aortic Aneurysms: Single Centre Results.* Eur J Vasc Endovasc Surg, 2015. **50**(4): p. 432-41.
103. Sheu, E.G., et al., *A comparative trial of laparoscopic magnetic sphincter augmentation and Nissen fundoplication.* Surg Endosc, 2015. **29**(3): p. 505-9.
104. Shute, J.B., et al., *Thalamocortical network activity enables chronic tic detection in humans with Tourette syndrome.* Neuroimage Clin, 2016. **12**: p. 165-72.
105. Soejima, K., et al., *Performance of Leadless Pacemaker in Japanese Patients vs. Rest of the World - Results From a Global Clinical Trial.* Circ J, 2017. **81**(11): p. 1589-1595.
106. Soejima, K., et al., *Safety evaluation of a leadless transcatheter pacemaker for magnetic resonance imaging use.* Heart Rhythm, 2016. **13**(10): p. 2056-63.
107. Sriratanaviriyakul, N., et al., *LINX®, a novel treatment for patients with refractory asthma complicated by gastroesophageal reflux disease: a case report.* J Med Case Rep, 2016. **10**(1): p. 124.
108. Starnes, B.W., *Physician-modified endovascular grafts for the treatment of elective, symptomatic, or ruptured juxtarenal aortic aneurysms.* J Vasc Surg, 2012. **56**(3): p. 601-7.
109. Swann, N.C., et al., *Chronic multisite brain recordings from a totally implantable bidirectional neural interface: experience in 5 patients with Parkinson's disease.* J Neurosurg, 2018. **128**(2): p. 605-616.
110. Swann, N.C., et al., *Adaptive deep brain stimulation for Parkinson's disease using motor cortex sensing.* J Neural Eng, 2018. **15**(4): p. 046006.
111. Syrkin-Nikolau, J., et al., *Subthalamic neural entropy is a feature of freezing of gait in freely moving people with Parkinson's disease.* Neurobiol Dis, 2017. **108**: p. 288-297.
112. Tapping, C.R., et al., *Three-year follow-up of fenestrated thoracoabdominal stent graft bridging an endovascular thoracic stent graft and a surgical abdominal aortic graft.* J Vasc Interv Radiol, 2011. **22**(3): p. 385-90.
113. Tapping, C.R., M.W. Little, and P. Boardman, *Successful Prostatic Artery Embolization following UroLift Device Failure.* J Vasc Interv Radiol, 2017. **28**(9): p. 1275-1276.
114. Tatum, J.M., et al., *Minimal versus obligatory dissection of the diaphragmatic hiatus during magnetic sphincter augmentation surgery.* Surg Endosc, 2019. **33**(3): p. 782-788.
115. Timaran, D.E., et al., *Fenestrated endovascular aneurysm repair among octogenarians at high and standard risk for open repair.* J Vasc Surg, 2017. **66**(2): p. 354-359.
116. Timaran, D.E., et al., *Safety and effectiveness of total percutaneous access for fenestrated endovascular aortic aneurysm repair.* J Vasc Surg, 2016. **64**(4): p. 896-901.
117. Trager, M.H., et al., *Subthalamic beta oscillations are attenuated after withdrawal of chronic high frequency neurostimulation in Parkinson's disease.* Neurobiol Dis, 2016. **96**: p. 22-30.
118. Ugurlucan, F.G., et al., *Randomized trial of graft materials in transobturator tape operation: biological versus synthetic.* Int Urogynecol J, 2013. **24**(8): p. 1315-23.
119. Ultee, K.H.J., et al., *Perioperative outcome of endovascular repair for complex abdominal aortic aneurysms.* J Vasc Surg, 2017. **65**(6): p. 1567-1575.
120. Van Gompel, J.J., et al., *Anterior nuclear deep brain stimulation guided by concordant hippocampal recording.* Neurosurg Focus, 2015. **38**(6): p. E9.
121. Vemuri, C., et al., *Postapproval outcomes of juxtarenal aortic aneurysms treated with the Zenith fenestrated endovascular graft.* J Vasc Surg, 2014. **60**(2): p. 295-300.
122. Vidya, R. and S.J. Cawthorn, *Muscle-Sparing ADM-Assisted Breast Reconstruction Technique Using Complete Breast Implant Coverage: A Dual-Institute UK-Based Experience.* Breast Care (Basel), 2017. **12**(4): p. 251-254.
123. Vidya, R., et al., *Evaluation of the effectiveness of the prepectoral breast reconstruction with Braxon dermal matrix: First multicenter European report on 100 cases.* Breast J, 2017. **23**(6): p. 670-676.


For peer review only

124. Waln, O. and J. Jimenez-Shahed, *Rechargeable deep brain stimulation implantable pulse generators in movement disorders: patient satisfaction and conversion parameters.* Neuromodulation, 2014. **17**(5): p. 425-30; discussion 430.
125. Wang, S.K., et al., *Successful Treatment of an Infected Zenith Fenestrated Endograft Without Explantation.* Vasc Endovascular Surg, 2018. **52**(7): p. 569-572.
126. Warren, H.F., et al., *Factors influencing the outcome of magnetic sphincter augmentation for chronic gastroesophageal reflux disease.* Surg Endosc, 2018. **32**(1): p. 405-412.
127. Wooster, M., et al., *Concomitant Parallel Endografting and Fenestrated Experience in a Regional Aortic Center.* Ann Vasc Surg, 2017. **38**: p. 54-58.
128. Zeidler, K.R., et al., *AeroForm patient controlled tissue expansion and saline tissue expansion for breast reconstruction: a randomized controlled trial.* Ann Plast Surg, 2014. **72 Suppl 1**: p. S51-5.

For peer review only

BMJ Open

# Identification of outcomes to inform the development of a core outcome set for surgical innovation: a targeted review of case studies of novel surgical devices



| | |
|---|---|
| Journal: | *BMJ Open* |
| Manuscript ID | bmjopen-2021-056003.R1 |
| Article Type: | Original research |
| Date Submitted by the Author: | 31-Jan-2022 |





| | |
|---|---|
| Complete List of Authors: | Wilson, Nicholas; University of Bristol, Population Health Sciences<br>Macefield, Rhiannon; University of Bristol, Population Health Sciences<br>Hoffmann, Christin; University of Bristol<br>Edmondson, Matthew J; Royal United Hospitals Bath NHS Foundation Trust<br>Miller, Rachael Lucia; University of Bristol, Population Health Sciences; North Bristol NHS Trust, Department of Surgery<br>Kirkham, Emily N; Gloucestershire Hospitals NHS Foundation Trust<br>Blencowe, Natalie; University of Bristol, Population Health Sciences<br>McNair, Angus; University of Bristol, Population Health Sciences; North Bristol NHS Trust, GI Surgery<br>Main, Barry; University of Bristol, Population Health Sciences; University Hospitals Bristol and Weston NHS Foundation Trust, Division of Surgery<br>Blazeby, Jane; University of Bristol, Department of Social Medicine<br>Avery, Kerry; University of Bristol, Population Health Sciences<br>Potter, Shelley; University of Bristol, Population Health Sciences; North Bristol NHS Trust, Bristol Breast Care Centre |





| | |
|---|---|
| <b>Primary Subject Heading</b>: | Surgery |
| Secondary Subject Heading: | Health services research, Research methods |
| Keywords: | STATISTICS & RESEARCH METHODS, SURGERY, PUBLIC HEALTH |
| | |



SCHOLARONE™ Manuscripts

*I, the Submitting Author has the right to grant and does grant on behalf of all authors of the Work (as defined in the below author licence), an exclusive licence and/or a non-exclusive licence for contributions from authors who are: i) UK Crown employees; ii) where BMJ has agreed a CC-BY licence shall apply, and/or iii) in accordance with the terms applicable for US Federal Government officers or employees acting as part of their official duties; on a worldwide, perpetual, irrevocable, royalty-free basis to BMJ Publishing Group Ltd ("BMJ") its licensees and where the relevant Journal is co-owned by BMJ to the co-owners of the Journal, to publish the Work in this journal and any other BMJ products and to exploit all rights, as set out in our licence.*

*The Submitting Author accepts and understands that any supply made under these terms is made by BMJ to the Submitting Author unless you are acting as an employee on behalf of your employer or a postgraduate student of an affiliated institution which is paying any applicable article publishing charge ("APC") for Open Access articles. Where the Submitting Author wishes to make the Work available on an Open Access basis (and intends to pay the relevant APC), the terms of reuse of such Open Access shall be governed by a Creative Commons licence – details of these licences and which Creative Commons licence will apply to this Work are set out in our licence referred to above.*

*Other than as permitted in any relevant BMJ Author's Self Archiving Policies, I confirm this Work has not been accepted for publication elsewhere, is not being considered for publication elsewhere and does not duplicate material already published. I confirm all authors consent to publication of this Work and authorise the granting of this licence.*

For peer review only

# Identification of outcomes to inform the development of a core outcome set for surgical innovation: a targeted review of case studies of novel surgical devices

Nicholas Wilson PhD[1]*, Rhiannon C Macefield PhD[1]*, Christin Hoffmann PhD[1], Matthew J Edmondson[2], Rachael L Miller[1,3] MRes, Emily N Kirkham[4], Natalie S Blencowe PhD[1,5], Angus GK McNair PhD[1,3], Barry G Main PhD [1,5], Jane M Blazeby MD[1,5], Kerry NL Avery PhD[1]**, Shelley Potter PhD[1,6]**

*joint first authors
**joint senior authors

**Affiliations:**

[1] National Institute for Health Research Bristol Biomedical Research Centre, Bristol Centre for Surgical Research, Bristol Medical School: Population Health Sciences, University of Bristol, 39 Whatley Road, Clifton, Bristol, BS8 2PS, UK

[2]Intensive Care, Royal United Hospitals Bath NHS Foundation Trust, BA1 3NG

[3] Department of Surgery, North Bristol NHS Trust, Southmead Road, Bristol, BS10 5NB

[4] Gloucestershire Hospitals NHS Foundation Trust, Great Western Road, Gloucester, GL1 3NN

[5] Division of Surgery, Bristol Royal Infirmary, University Hospitals Bristol and Weston NHS Foundation Trust, Bristol, BS2 8HW

[6] Bristol Breast Care Centre, North Bristol NHS Trust, Southmead Road, Bristol, BS10 5NB

**Corresponding author:**

Dr Rhiannon C Macefield, Bristol Centre for Surgical Research, University of Bristol, Canynge Hall, 39 Whatley Road, Clifton, Bristol, BS8 2PS, UK. Email: r.macefield@bristol.ac.uk

For peer review only



For peer review only - http://bmjopen.bmj.com/site/about/guidelines.xhtml

**Abstract**

**Objective:** Outcome selection and reporting in studies of novel surgical procedures and devices lacks standardisation, hindering safe and effective evaluation. A core outcome set (COS) to measure and report in all studies of surgical innovation is needed. We explored outcomes in a specific sample of innovative surgical device case studies to identify outcome domains specifically relevant to innovation to inform development of a COS.

**Design:** A targeted review of 11 purposive-selected case studies of innovative surgical devices.

**Methods:** Electronic database searches in PubMed (July 2018) identified publications reporting the introduction and evaluation of each device. Outcomes were extracted and categorised into domains until no new domains were conceptualised. Outcomes specifically relevant to evaluating innovation were further scrutinised.

**Results:** 112 relevant publications were identified, and 5,926 outcomes extracted. Heterogeneity in study type, outcome selection and reporting was observed across surgical devices. Categorisation of outcomes was performed for 2689 (45.4%) outcomes into five broad outcome domains. Outcomes considered key to the evaluation of innovation (n=66; 2.5%) were further categorised as surgeon/operator experience (n=40; 1.5%), unanticipated events (n=15, 0.6%), and modifications (n=11; 0.4%).

**Conclusion:** Outcome domains unique to evaluating innovative surgical devices have been identified. Findings have been combined with multiple other data sources relevant to the evaluation of surgical innovation to inform the development of a COS to measure and report in all studies evaluating novel surgical procedures/devices.

For peer review only

## Article summary

### Strengths and limitations of this study

- A purposive sample of surgical device case studies was selected to ensure broad representation across surgical specialties and disease areas.
- Studies were chosen to reflect the breadth of surgical device innovation.
- A broad definition of an outcome was adopted to ensure that all information potentially relevant to evaluating device innovation was captured.
- This review was limited by searching for relevant publications using device trade name only and restricting to English language publications.

For peer review only

## Introduction

The introduction and evaluation of innovative surgical procedures and devices is less regulated and standardised than that required for new pharmaceuticals. This can cause patient harm, as demonstrated by examples such as the introduction of metal-on-metal hip replacements and vaginal mesh implants [1, 2]. One particular challenge for the evaluation of surgical innovation is the lack of standardisation in the selection, measurement and reporting of outcomes in studies of new procedures and devices, limiting data synthesis and the ability to accurately compare and contrast device efficacy [3]. The IDEAL (Idea, Development, Exploration, Assessment, Long-term Follow-up) framework describes a structure and recommended study designs for the introduction and evaluation of new surgical procedures and devices [4, 5]. A consensus-agreed core set of outcomes that are essential to measure at each stage of the innovation pathway, however, is lacking from the guidance. Decisions regarding the choice of outcomes are currently made by study investigators. Outcomes may be selected *a priori* (based on hypothesised efficacy) or *post hoc* (based on statistical significance), rather than being selected because they are meaningful to patients or valuable to surgeon-innovators and other stakeholders looking to adopt the procedure. These issues risk the potential for introducing outcome reporting bias and inconsistent and heterogenous outcome selection which, when combined with thwarted data synthesis, may delay identification of the benefits and harms of new interventions [6].

A potential solution to this problem is to develop and use a core outcome set (COS) to evaluate innovative surgical procedures/devices. A core outcome set is an agreed set of outcomes to be measured and reported, as a minimum, in all studies in a particular area [7]. Use of COS have been shown to be effective at improving the quality and consistency of outcome selection and reporting in comparative effectiveness research, for example, randomised controlled trials (RCTs) [8]. Methods for developing COS for effectiveness studies for particular diseases or conditions are well-established. This includes, firstly, the identification of a 'long list' of all potential outcomes to inform the consensus process and reduce to a core set, typically by undertaking systematic reviews of RCTs in the specific disease or condition to identify outcomes of interest [7]. This is frequently followed by a Delphi consensus process involving key stakeholders to prioritise a minimum set of core outcomes that are essential to include in the COS. However, systematic reviews of RCTs are unlikely to identify outcomes of specific relevance to surgical innovation. Furthermore, important 'drivers' (that is, factors that may influence the development) of innovation may not be recognised and reported as outcomes in the traditional sense and instead may be reported in more descriptive terms. Novel approaches are needed. We hypothesised that publications reporting the introduction and evaluation of new surgical procedures and devices may generate specific insights into outcome selection and reporting in surgical



For peer review only - http://bmjopen.bmj.com/site/about/guidelines.xhtml

innovation, and thereby serve as valuable data sources to generate a long list of potential outcomes to inform the development of a future COS. The intention of this initial targeted review was to focus on surgical devices.

The aim of this study was to i) examine outcome selection and reporting in a purposive sample of case studies of known innovative surgical devices, ii) to categorise outcomes into domains; identifying outcomes specifically relevant to innovation and iii) conceptualise innovation domains for inclusion in a future Delphi consensus process to develop a COS [9, 10]. This study, focusing on studies of surgical devices, served as the first of multiple other data sources of relevance to surgical innovation to conceptualise outcome domains for inclusion in the COS [9].

## Methods

A targeted review of purposively-selected case studies of innovative surgical devices was undertaken. A purposive sampling approach (detailed below) was undertaken with the aim of representing, and being able to examine in depth, a broad and varied range of innovative surgical devices.

### *Selection of case studies*

The purposive sampling strategy was discussed and agreed amongst members of the multi-disciplinary study team. Specialists from a range of medical disciplines (orthopaedic surgery, neurosurgery, plastic surgery, gynaecology, gastrointestinal surgery, breast surgery, cardiology, urology, vascular surgery, maxillofacial surgery and ophthalmology) known to the study team were contacted and asked to identify innovative surgical devices developed within the last decade. A purposive sample of the identified devices were then selected by the study team to ensure broad representation across the surgical specialties, disease areas and “degree of novelty” of the innovation, to ensure that case studies reflected the breadth of the surgical device innovation lifecycle. Degree of novelty was categorised through discussion within the study team as ‘wholly innovative’ (a device representing a completely new approach to solving a clinical problem); ‘partially innovative’ (a device broadly similar in function to one already in use but differing in at least one significant way); ‘reinvented’ (a modification of a device or technique that had been previously been used but that was abandoned due to complications) or ‘predicate’ (a device introduced via the U.S. Food and Drug Administration (FDA) predicate 510(k) pathway on the basis of equivalence [11]).



For peer review only - http://bmjopen.bmj.com/site/about/guidelines.xhtml

*Identification and selection of publications*

Electronic searches were performed in PubMed to identify publications reporting the introduction and evaluation of each device in July 2018. Searches were performed using the tradename of the device as a text word (supplementary file 1). No publication date limits were applied. Search results were imported into Excel. Records were screened for eligibility by one reviewer with 10 per cent independently checked for eligibility by a second reviewer. Included were primary studies reporting the use of the device at any stage of development from first in human up to and including RCTs. Excluded were studies involving animals and publications in non-English languages. Letters and conference proceedings were also excluded due to lack of detail to extract comprehensive information on outcome selection.

*Data extraction*

Descriptive characteristics of included publications, including publication year, study details (type of study, design, number of centers involved and whether they had ethical/institutional board approval) and geographical origin, were extracted. Outcome data extraction for each publication was approached systematically starting with the title. Outcomes were extracted verbatim on first mention in the publication. Data extraction included generic descriptions of outcomes (for example, safety outcomes) as well as detailed, specific descriptions (for example, specific types of adverse events). For the purpose of this review, a broad definition of an outcome was adopted to ensure that all information potentially relevant to the evaluation of innovation was captured. This included any measured or reported construct or concept relating to or occurring as a result of using the device [6]. It also included descriptions or details of events and observations that occurred during the course of the study. Outcomes that were discussed (but not necessarily measured and reported as a study endpoint) by the publication's authors as being of interest or of relevance to evaluating the device were also extracted.

For each device case study, data extraction was performed on subsequent publications until no new outcomes were seemingly being observed. The order in which publications were selected for data extraction within each case study was not specified, irrelevant of publication date or study type. Data extraction was performed by one reviewer (either NW, RM, KA, RLM, EK or ME) with double data extraction completed on 10% of papers. Any discrepancies were discussed and resolved within the study team. Data were extracted directly into an electronic purpose-designed database using REDCap (Research Electronic Data Capture) software [12].



For peer review only - http://bmjopen.bmj.com/site/about/guidelines.xhtml

*Data analysis: categorisation of outcomes into domains*

The complete list of verbatim outcomes extracted from all publications was reviewed by members of the study team with clinical knowledge and methodological expertise in COS development (SP, KA, RM, NW). Outcomes were categorised into a conceptual framework of outcome domains (broad classifications of aspects relating to the effects or use of the device), referring back to source publications for context if needed. The conceptual framework of outcome domains was informed by the experience of the study team and knowledge of the existing taxonomies of outcomes traditionally measured in effectiveness studies [13]. During the progression of outcome categorisation, the framework of conceptualised domains was iteratively refined, informed by the data and discussion within the study team. Domains included, for example, adverse events (that is, unfavourable signs, symptoms or incidents), clinical efficacy (for example, indications of success in treating the condition) and patient-reported outcomes. During analysis, outcomes that were considered as specific to the evaluation of innovation in the context of the included case studies (that is, were particularly relevant for considering the future use, modification or uptake/abandonment of the device) were categorised as "key to evaluation of innovation". Categorisation of outcomes into domains continued until the list of domains was comprehensive and it was possible to categorise all outcomes into the conceptualised domains. For the purpose of this review, outcomes that were categorised as "key to evaluation of innovation" were scrutinised in more detail by the study team and further categorised into sub-domains, referring to the original publication for verification of context. These sub-domains were not predefined, and categorisation was iterative and data led.

Findings were summarised using descriptive statistics, where appropriate, and narrative summaries of the outcome domains relevant to evaluating innovation.

**Patient and Public Involvement**

This review was one component of a wider study to develop a COS for surgical innovation which sought patient and public input in the concept, design, analysis and dissemination at all stages of the study[9]. This was achieved through study steering group meetings with patient and public representatives and through two NIHR Bristol Biomedical Research Centre Surgical Innovation theme patient advisory group meetings. Patients and the public were not directly involved in conducting the review or performing the analysis.



For peer review only - http://bmjopen.bmj.com/site/about/guidelines.xhtml

## Results

*Device case studies and search results*

Eleven device case studies were identified to reflect the breadth of the device innovation lifecycle. Details of each device, their degree of novelty and brief descriptions of their functions are provided in Table 1.

Electronic database searches yielded 242 records, of which 114 (47%) were excluded (Figure 1). Full-text articles of the remaining 128 records were examined for eligibility. All reported on the use and/or evaluation of the device under study and were included for review (supplementary file 2).

*Data extraction*

In total, data extraction was completed on 112/128 (87.5%) of all included publications (Figure 1). This included all publications for 10 of the 11 device case studies. The exception was the Activa Deep Brain Stimulation device. This was the last case study for which data extraction was conducted. After data extraction from 11 of the 27 included publications no new outcomes were observed to be emerging and the study team agreed that further extraction would not likely add to the conceptualisation of outcome domains. The remaining 16 publications identified for this case study were, therefore, excluded from further analysis.

*Publication characteristics*

Descriptive details of the analysed publications, presented by device case study, are displayed in Table 2. Publication dates ranged from 2009 to 2018. Most publications were from North America (n=49; 43.8%), followed by the European Economic Area (n=25; 22.3%). Publications were predominantly case series including non-comparative cohort studies (n=60; 53.6%). Other publications were single patient case reports (n=24; 21.4%), non-randomised comparative studies (n=19; 17.0%) and a small number of RCTs (n=9; 8%). Just over half of the publications reported data from a single center (n=61; 54.5%) and approximately a third reported data from multi-center studies (n=40; 35.7%). Some 11 publications (9.8%) did not report the number of centers involved. Publications reported retrospective (n=62; 55.0%) and prospective (n=50; 45.0%) data collection. A statement confirming ethical or institutional board review approval was reported in 72 (64.3%) publications.



For peer review only - http://bmjopen.bmj.com/site/about/guidelines.xhtml

*Categorisation of outcomes into domains*

A total of 5,926 outcomes were extracted from the 112 included publications (Table 2). The number of extracted outcomes for each device case study ranged from 117 individual outcomes (Magseed device; extracted from a total of two publications) to 1,320 outcomes (LINX reflux Management System; extracted from a total of 22 publications). The median number of outcomes extracted from each unique publication was 48, ranging from five to 135.

Outcome categorisation continued until 2689 (45.4%) outcomes had been categorised to derive a conceptual framework of five broad outcome domains. The conceptual framework included: 1) anticipated or procedural events (defined as expected or routine incidents or consequences, n=1192; 44.3%), 2) clinical, technical or common data element parameters (defined as routinely-measured data variables of interest to the clinical specialty [14], n=745; 27.7%), 3) device function (defined as technical or operational performance parameters specific to the device, n=446; 16.6%), 4) patient-reported outcomes (n= 240; 8.9%,) and; 5) outcomes key to evaluating innovation (that is, considered to be particularly relevant to the future use, modification or uptake/abandonment of the device, n=66, 2.5%) (Figure 2). The remaining 3237 (54.6%) outcomes were not categorised as the list of conceptualised domains was agreed by the study team to be comprehensive and exhaustive (that is, it was possible to categorise subsequent outcomes into the existing domains) or because outcomes were too broad in their description to make categorisation possible (for example “safety”).

*Outcomes key to evaluating innovation*

Outcomes categorised as key to the evaluation of innovation (n=66; 2.5%) were further categorised into three sub-domains: surgeon/operator experience, unanticipated events, and modifications.

Some 40 (1.5%) outcomes were categorised as relating to surgeon/operator experience. These included, for example, ease of using the new device, convenience and satisfaction. For the majority of the included case studies, the operator of the device was the surgeon. In the case study of the AeroForm tissue expander, the device was operated by the patient following discharge from hospital. An example of an outcome relevant to patients’ experience of operating the device included “very easy” use of the dosage controller [15].

Some 15 (0.6%) outcomes were categorised as unanticipated events. These included adverse events related to the use of the device that were reported by the authors as unexpected or not previously



For peer review only - http://bmjopen.bmj.com/site/about/guidelines.xhtml

encountered or anticipated. An example of an outcome coded as an unanticipated event was an inability to dislodge the device in a retrieval attempt in the case of the leadless cardiac pacemaker (Micra LCP) [16]. A further example included fatal intestinal injury during surgery in the case study of the Align urethral support system [17].

Some 11 (0.4%) outcomes were categorised as modifications. These included adjustments to the device, for example, redesign of the internal membrane of the AeroForm tissue expander following testing during its development [18]. Authors noted how the expander bulk was decreased to minimise the risk of underexpansion and erosion, and therefore potential harm, in subsequent patients [18]. Detail on modification outcomes, however, were rarely reported in any of the other device case studies. Of the 11 modification outcomes, seven were from the Aeroform case study with the remaining four extracted from only three of the other case studies. Modifications were often described by authors in general terms and without specific descriptions, such as "design modifications" in the example of the Accolade hip stem [19] and "modifications to the liner" or "midtrial modifications to the expander" in the example of the AeroForm device [20].

**Discussion**

This review identified a broad range of outcomes identified, selected and reported in studies describing the introduction and evaluation of a purposive sample of novel devices, chosen to reflect the breadth of surgical device innovation. Although relatively few in number, outcomes considered key to the evaluation of innovation were identified from these empirical case studies, distinct from most outcomes shared with effective studies [13] . These were broadly categorised into outcome domains 'surgeon/operator experience', 'unanticipated events' and 'modifications'. Although infrequently reported in the studies included in this review, we consider these types of outcomes to be important drivers of innovation at the early stage of the device lifecycle and key to assessing procedures with new medical devices.

Recent systematic reviews have demonstrated that outcome reporting in studies of innovative surgical procedures and devices focus on short term, clinical and technical outcomes [21, 22]. These outcomes are critical to assess in any early phase study and are required for US Food and Drug Administration (FDA) and the UK Medicines and Healthcare products Regulatory Agency (MHRA) approval [23, 24].



For peer review only - http://bmjopen.bmj.com/site/about/guidelines.xhtml

These types of outcomes are also the recommended focus of early-phase studies (stages 1 and 2) in the IDEAL framework for the introduction and evaluation of new surgical procedures, and devices (IDEAL-D) [4, 5]. The current review, however, has studied outcome selection and reporting in depth in empirical case studies, in order to identify innovation-specific outcomes potentially important to evaluating the process of innovation that have not explicitly been specified as key outcomes in existing guidance. Assessing surgeon/operator experience of using a new device, for example, can highlight issues and provide valuable feedback for the device developers and clinical colleagues, playing an important role in making improvements in future versions of the device or the way in which the device is inserted or used. User experience may also determine whether a device is adopted into practice or abandoned, further underlining the relevance of this outcome as a key driver to innovation. The examples of unanticipated events identified in this review are also vitally important to the introduction and evaluation of innovation as they may represent patient safety issues that should be shared with clinicians undertaking the procedure in the future to minimise patient harm. It is recognised that unanticipated events are commonly reported in later-phase effectiveness studies such as RCTs, in particular in studies evaluating pharmaceuticals. In the context of early-phase studies of surgical devices, however, they are particularly important to highlight unexpected problems requiring modifications to the device or the procedure for using or inserting it. Such modifications may improve the efficacy of the device, its ease of use or, perhaps most importantly, reduce the risk of patient harm. Reporting unanticipated outcomes and modifications in early stages of the introduction of a device is key to shared learning relating to innovation with the wider surgical community, providing vital information to surgeons using the device in the future and to avoid repeated mistakes or harms.

This review examined a broad range of case studies of novel devices across the innovation lifecycle and across different surgical specialties. There are, however, some limitations. Case studies were identified by asking specialists known to the study team rather than a wider survey. A purposively-selected sample covering a range of specialties and stage of innovation were subsequently included. It is acknowledged that the 11 included case studies are not an exhaustive sample and may not be representative of all innovative devices. This study, however, was one of many data sources to inform the list of outcomes to consider for development of the COS for surgical innovation, which used triangulated data from published literature, qualitative interviews and regulatory body documents that focused on surgical procedures and/or devices [9]. Identification of publications involved database searches performed using the device trade name as the key search term. It is recognised that some relevant studies conducted before the trade name was established may have been missed. It is also acknowledged that a small number of outcomes may have been missed during data



For peer review only - http://bmjopen.bmj.com/site/about/guidelines.xhtml

extraction, with only 10% of papers undergoing a second reviewer independent check. It is unlikely, however, that this would have altered the conceptualised framework of outcome domains. During analysis, a broad approach to categorise outcomes was taken, using a conceptual framework that evolved during the iterative process of data analysis. Categorisation of outcomes during the earlier stages of analysis, therefore, may not represent how they may have been defined later in the evolution of the conceptual framework. Furthermore, each outcome was categorised only to a single outcome domain. For some outcomes, more than one domain many have been applicable, for example, "complete tumor excision" could be categorised as a procedural event or a clinical parameter. Decisions were based on reviewers' judgement which limits the reproducibility of the exact number of outcomes categorised to each domain. Outcomes categorised as key to evaluation of innovation were, however, scrutinised in more detail by referring to the contextual information in the original publication to better understand the nature of the outcome. Furthermore, the aim of this review was to identify outcomes and domains of specific relevance to innovation and the methodology used was successful in achieving this goal. Finally, this review only included case studies of novel surgical devices. These were specifically selected to identify outcomes that may have relevance to the introduction and evaluation of medical devices, hypothesising that there may be innovation outcomes specific to device use. By focusing on devices, this review may have failed to identify key innovation outcomes of relevance to the introduction of surgical techniques not involving a device. This review, however, was only one of multiple data sources used to inform the list of outcomes for later COS development [9, 25, 26].

Improvements in the processes for the introduction and evaluation of surgical innovations are a recognised need. The recent UK Independent Medicines and Medical Devices Safety Review, commissioned after the catastrophic consequences and harms experienced by some patients, highlighted the gaps and inefficiencies in the current regulatory systems [27]. Our work has explored the complexities surrounding the introduction of new procedures/devices into routine practice, highlighting shortcomings and inconsistencies in how surgical innovation is evaluated and reported [21, 22, 28]. This has highlighted the need for consensus on the outcomes that are essential to measure to support the safe and efficient introduction and evaluation of new surgical procedures and devices.

Findings from the current study, combined with outcomes identified from other data sources [25, 26] including reviews of studies of surgical procedures, interviews with surgeons and regulatory body



For peer review only - http://bmjopen.bmj.com/site/about/guidelines.xhtml

documents to inform an international Delphi consensus process with key stakeholders to agree on a core set of outcomes for use in all studies evaluating new surgical procedures/devices (the COHESIVE core outcome set) [10]. Mandatory measurement and reporting of this core outcome set in all future studies of new surgical procedures and devices will ensure that key outcomes of relevance to innovation that are important to patients and key stakeholder are included. The aim of the COS is to improve standardisation and consistency across studies, allowing results to be compared and combined. Furthermore, reporting and dissemination of these outcomes in real-time would inform surgeons, patients, device manufacturers and other key stakeholders of both the benefits and harms of new surgical procedures/devices in a timely manner and promote their safe, transparent and efficient evaluation and introduction and into clinical practice, avoiding patient harms. Work is currently underway to encourage the effective uptake of the new COS, to identify how to best measure these novel outcome domains (i.e. metrics and timing of assessments), and to develop a real-time reporting and sharing platform to streamline the process of surgical innovation to support surgeon-innovators and improve patient outcomes.

For peer review only



For peer review only - http://bmjopen.bmj.com/site/about/guidelines.xhtml

## Footnotes

**Author contributions:** NW and RCM participated in the research design, performance of the research, data analysis and writing of the paper; CH participated in the performance of the research and data analysis; ME, RLM, EK participated in the performance of the research; NB, AM and BM participated in the intellectual content, research design and performance of the research; JB participated in the intellectual content, research design and writing of the paper, KA and SP participated in the intellectual content, research design, the performance of the research, data analysis and writing of the paper.

**Funding:** This study was funded by the National Institute for Health Research (NIHR) Biomedical Research Centre (BRC) at University Hospitals Bristol and Weston NHS Foundation Trust and the University of Bristol (BRC-1215-20011). This work was supported by the Royal College of Surgeons of England Bristol Surgical Trials Centre, the MRC ConDuCT-II (Collaboration and innovation for Difficult and Complex randomised controlled Trials In Invasive procedures) Hub for Trials Methodology Research (MR/K025643/1) and the MRC-NIHR Trials Methodology Research Partnership (TMRP). SP and AM are NIHR Clinician Scientists (NIHR CS-2016-16-019, NIHR CS-2017-17-010). NB is an MRC Clinician Scientist. BGM is a NIHR Clinical Lecturer. JB is an NIHR Senior Investigator. The views and opinions expressed in this publication are those of the authors and not necessarily those of the National Institute for Health Research, the Department of Health and Social Care, the Royal College of Surgeons of England or the Medical Research Council. The authors declare no conflicts of interest.

**Competing interests:** None declared.

**Data availability statement:** All data relevant to the study are included in the article or uploaded as supplementary information.

**Ethics statement:** This study does not involve human participants.

For peer review only

**References**


1. Cohen, D., *How safe are metal-on-metal hip implants?* British Medical Journal, 2012. **344**: p. e1410.
2. Heneghan, C., et al., *Transvaginal mesh failure: lessons for regulation of implantable devices.* British Medical Journal, 2017. **359**: p. j5515.
3. Ergina, P.L., et al., *Surgical Innovation and Evaluation 2 Challenges in evaluating surgical innovation.* Lancet, 2009. **374**(9695): p. 1097-1104.
4. McCulloch, P., et al., *No surgical innovation without evaluation: the IDEAL recommendations.* Lancet, 2009. **374**(9695): p. 1105-12.
5. Sedrakyan, A., et al., *IDEAL-D: a rational framework for evaluating and regulating the use of medical devices.* BMJ, 2016. **353**: p. i2372.
6. Gorst, S.L., et al., *Choosing Important Health Outcomes for Comparative Effectiveness Research: An Updated Review and Identification of Gaps.* PLOS ONE, 2016. **11**(12): p. e0168403.
7. Williamson, P.R., et al., *The COMET Handbook: version 1.0.* Trials, 2017. **18**(Suppl 3): p. 280.
8. Tunis, S.R., et al., *Improving the relevance and consistency of outcomes in comparative effectiveness research.* J Comp Eff Res, 2016. **5**(2): p. 193-205.
9. Avery, K., et al., *Development of reporting guidance and core outcome sets for seamless, standardised evaluation of innovative surgical procedures and devices: a study protocol for content generation and a Delphi consensus process (COHESIVE study).* BMJ Open, 2019. **9**(9): p. e029574.
10. Avery, K., et al., *A core outcome set for seamless, standardised evaluation and reporting of outcomes throughout the surgical innovation lifecycle: the COHESIVE study.* British Journal of Surgery, 2020. **107**(S4): p. 11-37.
11. US Food and Drug Administration. *Premarket Notification 510(k)*. Accessed on 07/12/2020]; Available from: https://www.fda.gov/medical-devices/premarket-submissions/premarket-notification-510k.
12. Harris, P.A., et al., *Research electronic data capture (REDCap) - A metadata-driven methodology and workflow process for providing translational research informatics support.* Journal of Biomedical Informatics, 2009. **42**(2): p. 377-81.
13. Dodd, S., et al., *A taxonomy has been developed for outcomes in medical research to help improve knowledge discovery.* J Clin Epidemiol, 2018. **96**: p. 84-92.
14. Karaa, A., et al., *Common data elements for clinical research in mitochondrial disease: a National Institute for Neurological Disorders and Stroke project.* J Inherit Metab Dis, 2017. **40**(3): p. 403-414.
15. Connell, T.F., *Patient-activated controlled expansion for breast reconstruction using controlled carbon dioxide inflation: confirmation of a feasibility study.* Plast Reconstr Surg, 2014. **134**(4): p. 503e-11e.
16. Grubman, E., et al., *To retrieve, or not to retrieve: System revisions with the Micra transcatheter pacemaker.* Heart Rhythm, 2017. **14**(12): p. 1801-1806.
17. Kascak, P. and B. Kopcan, *Fatal Injury of the Small Intestine during Retropubic Sling Placement.* Case Rep Obstet Gynecol, 2015. **2015**: p. 164545.
18. Zeidler, K.R., et al., *AeroForm patient controlled tissue expansion and saline tissue expansion for breast reconstruction: a randomized controlled trial.* Ann Plast Surg, 2014. **72 Suppl 1**: p. S51-5.
19. Nam, D., et al., *An evaluation of proximal femur bone density in young, active patients undergoing total hip arthroplasty at one year postoperatively.* Hip Int, 2019. **29**(1): p. 51-57.
20. Ascherman, J.A., et al., *Carbon Dioxide-Based versus Saline Tissue Expansion for Breast Reconstruction: Results of the XPAND Prospective, Randomized Clinical Trial.* Plast Reconstr Surg, 2016. **138**(6): p. 1161-1170.


For peer review only



For peer review only - http://bmjopen.bmj.com/site/about/guidelines.xhtml

21. Pathak, S., et al., *A Systematic Review of Minimally Invasive Trans-thoracic Liver Resection to Examine Intervention Description, Governance, and Outcome Reporting of an Innovative Technique.* Ann Surg, 2020.
22. Kirkham, E.N., et al., *Systematic review of the introduction and evaluation of magnetic augmentation of the lower oesophageal sphincter for gastro-oesophageal reflux disease.* Br J Surg, 2020. **107**(1): p. 44-55.
23. US Food and Drug Administration. *Device Advice. Comprehensive Regulatory Assistance*. 28/01/2021]; Available from: https://www.fda.gov/medical-devices/device-advice-comprehensive-regulatory-assistance.
24. Medicines and Healthcare products Regulatory Agency. *Guidance - Medical devices: how to comply with the legal requirements* 2014 28/01/2021]; Available from: https://www.gov.uk/guidance/medical-devices-how-to-comply-with-the-legal-requirements.
25. Hoffmann, C., et al., *A systematic review and in-depth analysis of outcome reporting in early phase studies of colorectal cancer surgical innovation.* Colorectal Dis, 2020.
26. Macefield, R.C., et al., *Outcome selection, measurement and reporting for new surgical procedures and devices: a systematic review of IDEAL/IDEAL-D studies to inform development of a core outcome set.* BJS Open, 2020.
27. Cumberlege, J. *First Do No Harm: The report of the Independent Medicines and Medical Devices Safety Review*. 2020.
28. Zahra, J., et al., *Discussing surgical innovation with patients: a qualitative study of surgeons' and governance representatives' views.* BMJ Open, 2020. **10**(11): p. e035251.


For peer review only

**Figure 1.** Identification and selection of included publications

**Figure 2**. Categorisation of outcomes extracted from all publications (n=2689)

For peer review only



For peer review only - http://bmjopen.bmj.com/site/about/guidelines.xhtml

**Table 1.** Description of the 11 medical devices selected to explore outcome selection and reporting.

| Degree of novelty* | Device tradename | Licenced by | Surgical Specialty | Description of device |
|---|---|---|---|---|
| Wholly innovative | Activa Deep Brain Stimulation | Medtronic | Neurosurgery | A dual-channel, rechargeable neurostimulator for treatment of neurological conditions including Parkinson's disease, dystonia and essential tremor. The device is typically implanted in the chest or abdomen, connected to an extension and leads, which are implanted in the brain |
| | Magseed | Endomag | Breast | A ferromagnetic localisation seed used to localise impalpable breast lesions prior to excision. |
| | UroLift | Neotract Inc | Urology | A minimally invasive treatment of benign prostatic hyperplasia or an enlarged prostate. |
| Partially innovative | AeroForm | AirXpanders | Plastic/Breast | A remote-control tissue expander filled from an internal source with carbon dioxide eliminating the need for needle injections to fill the expander. This expander makes breast reconstruction, faster and more comfortable while providing women with some degree of control over the expansion. |
| | Micra leadless pacemaker | Medtronic | Cardiology | A pacemaker implanted directly into the patient's heart. |
| | Zenith fenestrated AAA graft | Cook Medical | Vascular | An endovascular graft indicated for the endovascular treatment of patients with abdominal aortic or aorto-iliac aneurysms of a morphology not suitable for standard endovascular repair. |
| Reinvented | BRAXON | Raise Healthcare | Plastic/Breast | A pre-shaped porcine acellular dermal matrix designed to completely enclose the breast implant prior to fixation on top of the intact pectoralis major muscle for breast reconstruction. |
| | LINX Reflux Management System | Ethicon (formally Torax Medical) | Gastrointestinal | An anti-reflux device that can be inserted laparoscopically around the lower end of the esophagus for the treatment of gastroesophageal reflux disease. |
| Predicate | ACCOLADE II hip stem | Stryker | Orthopaedics | A morphometric wedge femoral hip stem, merging conventional tapered wedge femoral stem design with size specific medial curvature to more closely fit a broad range of bone sizes and shapes of today's patient population. |
| | ALIGN Urethral Support System | Bard Medical | Urology/ Gynaecology | A macroporous lightweight polypropylene mesh designed to treat cases of stress urinary incontinence as well as pelvic organ prolapse |
| | BioDesign fistula plug | Cook Medical | Gastrointestinal | A fistula plug for implantation to reinforce soft tissue for the repair of recto-vaginal or anorectal fistulas. |

For peer review only

*Wholly innovative: a device that represents a completely new approach to solving a clinical problem; Partially innovative: a device that is broadly similar in function to one already in use but differs in at least one significant way; Reinvented: a modification of a device or technique that was previously abandoned for complications; Predicate: a device introduced via the FDA predicate 510(k) pathway on the basis of equivalence

For peer review only

**Table 2**. Publication characteristics and number of outcomes extracted for the 11 case studies of innovative medical devices.

| Degree of novelty* | | Wholly innovative | | | Partially innovative | | | Reinvented | | Predicate | | |
|---|---|---|---|---|---|---|---|---|---|---|---|---|
| | **Overall** | Activa Deep Brain Stimulation | Magseed | UroLift | AeroForm | Micra leadless pacemaker | Zenith fenestrated AAA graft | BRAXON | LINX Reflux Management System | ACCOLADE II hip stem | ALIGN Urethral Support System | BioDesign fistula plug |
| Number of publications included | **112** | 11 | 2 | 14 | 5 | 18 | 22 | 8 | 22 | 2 | 4 | 4 |
| Year of publication (range) | **2009-2018** | 2017-2018 | 2018 | 2013-2018 | 2014-2017 | 2015-2018 | 2016-2018 | 2016-2018 | 2010-2018 | 2014-2018 | 2013-2015 | 2009-2015 |
| Country of origin, n (%) | | | | | | | | | | | | |
| EEA | **25 (22.3)** | 3 | 0 | 2 | 0 | 3 | 4 | 4 | 5 | 0 | 2 | 2 |
| North America | **49 (43.8)** | 6 | 1 | 3 | 3 | 2 | 15 | 0 | 15 | 2 | 2 | 0 |
| UK | **12 (10.7)** | 1 | 1 | 3 | 0 | 1 | 2 | 3 | 0 | 0 | 0 | 1 |
| Multiple | **17 (15.2)** | 0 | 0 | 5 | 0 | 10 | 0 | 0 | 2 | 0 | 0 | 0 |
| Other | **9 (8.0)** | 1 | 0 | 1 | 2 | 2 | 1 | 1 | 0 | 0 | 0 | 1 |
| Type of publication, n (%) | | | | | | | | | | | | |
| Case report | **24 (21.4)** | 3 | 0 | 2 | 0 | 7 | 3 | 1 | 7 | 0 | 1 | 0 |
| Case series | **60 (53.6)** | 6 | 2 | 6 | 1 | 2 | 17 | 7 | 11 | 2 | 2 | 4 |
| Non-randomised comparative study | **19 (17.0)** | 2 | 0 | 1 | 2 | 9 | 2 | 0 | 3 | 0 | 0 | 0 |

For peer review only

| | | | | | | | | | | | | |
|---|---|---|---|---|---|---|---|---|---|---|---|---|
| Randomised controlled trial | **9 (8.0)** | 0 | 0 | 5 | 2 | 0 | 0 | 0 | 1 | 0 | 1 | 0 |
| Multi/single center, n (%) | | | | | | | | | | | | |
| Single center | **61 (54.5)** | 8 | 1 | 5 | 3 | 7 | 15 | 5 | 10 | 1 | 4 | 2 |
| Multicenter | **40 (35.7)** | 2 | 1 | 6 | 2 | 11 | 5 | 2 | 9 | 0 | 0 | 2 |
| Not reported | **11 (9.8)** | 1 | 0 | 3 | 0 | 0 | 2 | 1 | 3 | 1 | 0 | 0 |
| Data collection, n (%) | | | | | | | | | | | | |
| Retrospective | **62 (55)** | 6 | 0 | 8 | 0 | 8 | 15 | 4 | 15 | 0 | 3 | 3 |
| Prospective | **50 (45)** | 5 | 2 | 6 | 5 | 10 | 7 | 4 | 7 | 2 | 1 | 1 |
| Ethical /Institutional review board approved, n (%) | | | | | | | | | | | | |
| Yes | **72 (64.3)** | 7 | 2 | 7 | 3 | 10 | 14 | 4 | 17 | 2 | 3 | 3 |
| No | **40 (35.7)** | 4 | 0 | 7 | 2 | 8 | 8 | 4 | 5 | 0 | 1 | 1 |
| Number of outcomes extracted | **5,926** | 635 | 117 | 423 | 264 | 1,012 | 1,070 | 611 | 1,320 | 122 | 233 | 119 |

*Wholly innovative: a device that represents a completely new approach to solving a clinical problem; Partially innovative: a device that is broadly similar in function to one already in use but differs in at least one significant way; Reinvented: a modification of a device or technique that was previously abandoned for complications; Predicate: a device introduced via the FDA predicate 510(k) pathway on the basis of equivalence

For peer review only - http://bmjopen.bmj.com/site/about/guidelines.xhtml

**Figure 1.** Identification and selection of included publications

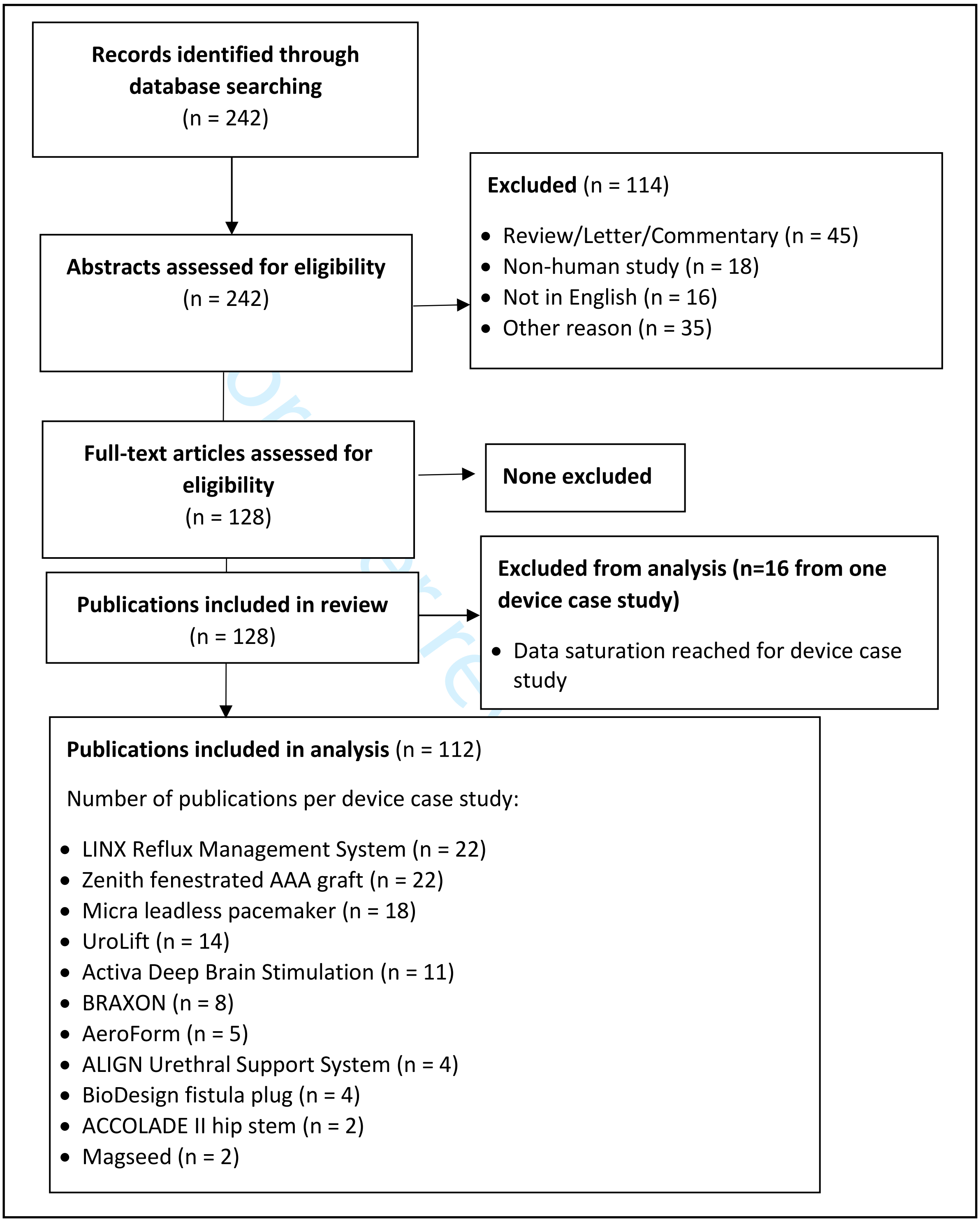

**Figure 2**. Categorisation of outcomes extracted from all publications (n=2689)

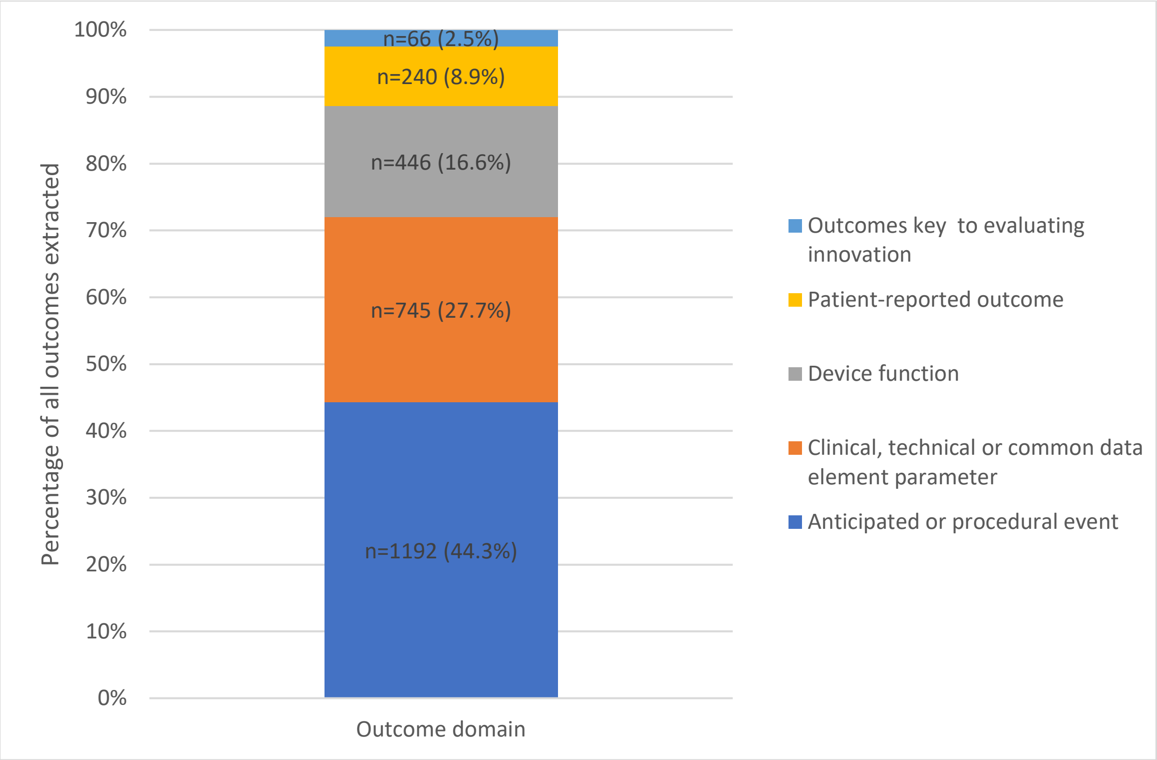


review only

Search strategy

All searches were performed in PubMed in July 2018.

Searches were performed by entering the device trade name into the search box with no other filters or limits used. This created and ran an automatically generated search query within the PubMed database. Two examples of the automatically generated search query for two of the device case studies are shown here:

**Accolade II**

"Accolade"[All Fields] AND "II"[All Fields]

**Braxon**

"Braxon"[All Fields]

For peer review only

Publications included in the review (n=128)


1. Afzal, M.R., et al., *Techniques for successful early retrieval of the Micra transcatheter pacing system: A worldwide experience.* Heart Rhythm, 2018. **15**(6): p. 841-846.
2. Ahmed, F.Z., et al., *Totally Leadless Dual-Device Implantation for Combined Spontaneous Ventricular Tachycardia Defibrillation and Pacemaker Function: A First Report.* Can J Cardiol, 2017. **33**(8): p. 1066.e5-1066.e7.
3. Alicuben, E.T., et al., *Worldwide Experience with Erosion of the Magnetic Sphincter Augmentation Device.* J Gastrointest Surg, 2018. **22**(8): p. 1442-1447.
4. Ascherman, J.A., et al., *Carbon Dioxide-Based versus Saline Tissue Expansion for Breast Reconstruction: Results of the XPAND Prospective, Randomized Clinical Trial.* Plast Reconstr Surg, 2016. **138**(6): p. 1161-1170.
5. Asti, E., et al., *Longitudinal comparison of quality of life in patients undergoing laparoscopic Toupet fundoplication versus magnetic sphincter augmentation: Observational cohort study with propensity score analysis.* Medicine (Baltimore), 2016. **95**(30): p. e4366.
6. Asti, E., et al., *Removal of the Magnetic Sphincter Augmentation Device: Surgical Technique and Results of a Single-center Cohort Study.* Ann Surg, 2017. **265**(5): p. 941-945.
7. Bardoli, A.D., W.S.J. Taylor, and W. Mahmalji, *Can the UroLift prostatic implant device treat the symptoms of benign prostatic hypertrophy, avoid sexual dysfunction and reduce hospital TURP waiting times? A single centre, single surgeon experience and review of the literature.* Aging Male, 2017. **20**(3): p. 192-197.
8. Bauer, M., et al., *Endoluminal perforation of a magnetic antireflux device.* Surg Endosc, 2015. **29**(12): p. 3806-10.
9. Bell, R., et al., *Laparoscopic magnetic sphincter augmentation versus double-dose proton pump inhibitors for management of moderate-to-severe regurgitation in GERD: a randomized controlled trial.* Gastrointest Endosc, 2019. **89**(1): p. 14-22.e1.
10. Berna, G., et al., *Evaluation of a novel breast reconstruction technique using the Braxon(®) acellular dermal matrix: a new muscle-sparing breast reconstruction.* ANZ J Surg, 2017. **87**(6): p. 493-498.
11. Biswal, N.C., et al., *UroLift as a surrogate for fiducial markers in IGRT planning of prostate cancer in BPH patients.* Pract Radiat Oncol, 2018. **8**(4): p. e231-e233.
12. Blom, J., et al., *Results of collagen plug occlusion of anal fistula: a multicentre study of 126 patients.* Colorectal Dis, 2014. **16**(8): p. 626-30.
13. Blumenfeld, Z., et al., *Sixty-hertz stimulation improves bradykinesia and amplifies subthalamic low-frequency oscillations.* Mov Disord, 2017. **32**(1): p. 80-88.
14. Bonavina, L., et al., *Laparoscopic sphincter augmentation device eliminates reflux symptoms and normalizes esophageal acid exposure: one- and 2-year results of a feasibility trial.* Ann Surg, 2010. **252**(5): p. 857-62.
15. Bozkurt, A., et al., *Prostatic Urethral Lift: A New Minimally Invasive Treatment for Lower Urinary Tract Symptoms Secondary to Benign Prostatic Hyperplasia.* Urol Int, 2016. **96**(2): p. 202-6.
16. Buckley, F.P., 3rd, et al., *Favorable results from a prospective evaluation of 200 patients with large hiatal hernias undergoing LINX magnetic sphincter augmentation.* Surg Endosc, 2018. **32**(4): p. 1762-1768.
17. Cantwell, A.L., et al., *Multicentre prospective crossover study of the 'prostatic urethral lift' for the treatment of lower urinary tract symptoms secondary to benign prostatic hyperplasia.* BJU Int, 2014. **113**(4): p. 615-22.
18. Chelvarajah, R., et al., *Shielded battery syndrome: a new hardware complication of deep brain stimulation.* Stereotact Funct Neurosurg, 2012. **90**(2): p. 113-7.
19. Chin, P. and P. Robertson, *Medium-term efficacy of the prostatic urethral lift.* Transl Androl Urol, 2017. **6**(Suppl 2): p. S122-s132.


For peer review only

20. Chinitz, L., et al., *Accelerometer-based atrioventricular synchronous pacing with a ventricular leadless pacemaker: Results from the Micra atrioventricular feasibility studies.* Heart Rhythm, 2018. **15**(9): p. 1363-1371.
21. Connell, T.F., *Patient-activated controlled expansion for breast reconstruction using controlled carbon dioxide inflation: confirmation of a feasibility study.* Plast Reconstr Surg, 2014. **134**(4): p. 503e-11e.
22. Connell, T.F., *Results from the ASPIRE study for breast reconstruction utilizing the AeroForm™ patient controlled carbon dioxide-inflated tissue expanders.* J Plast Reconstr Aesthet Surg, 2015. **68**(9): p. 1255-61.
23. de Souza, L.R., et al., *Editor's Choice - Comparison of Renal Outcomes in Patients Treated by Zenith(®) Fenestrated and Zenith(®) Abdominal Aortic Aneurysm Stent grafts in US Prospective Pivotal Trials.* Eur J Vasc Endovasc Surg, 2017. **53**(5): p. 648-655.
24. Desart, K., et al., *Gastroesophageal Reflux Management with the LINX® System for Gastroesophageal Reflux Disease Following Laparoscopic Sleeve Gastrectomy.* J Gastrointest Surg, 2015. **19**(10): p. 1782-6.
25. Dorsey, C., V. Chandra, and J.T. Lee, *The "terrace technique"--totally endovascular repair of a type IV thoracoabdominal aortic aneurysm.* Ann Vasc Surg, 2014. **28**(6): p. 1563.e11-6.
26. Duray, G.Z., et al., *Long-term performance of a transcatheter pacing system: 12-Month results from the Micra Transcatheter Pacing Study.* Heart Rhythm, 2017. **14**(5): p. 702-709.
27. El-Chami, M., et al., *Impact of operator experience and training strategy on procedural outcomes with leadless pacing: Insights from the Micra Transcatheter Pacing Study.* Pacing Clin Electrophysiol, 2017. **40**(7): p. 834-842.
28. England, A., et al., *Migration of fenestrated aortic stent grafts.* J Vasc Surg, 2013. **57**(6): p. 1543-52.
29. England, A., M. García-Fiñana, and R.G. McWilliams, *Multicenter retrospective investigation into migration of fenestrated aortic stent grafts.* J Vasc Surg, 2015. **62**(4): p. 884-92.
30. Farber, M.A., R. Vallabhaneni, and W.A. Marston, *"Off-the-shelf" devices for complex aortic aneurysm repair.* J Vasc Surg, 2014. **60**(3): p. 579-84.
31. Filgate, R., A. Thomas, and M. Ballal, *Treatment of foregut fistula with biologic plugs.* Surg Endosc, 2015. **29**(7): p. 2006-12.
32. Fisher, B., et al., *Battery Longevity Comparison of Two Commonly Available Dual Channel Implantable Pulse Generators Used for Subthalamic Nucleus Stimulation in Parkinson's Disease.* Stereotact Funct Neurosurg, 2018. **96**(3): p. 151-156.
33. Gallitto, E., et al., *Results of standard suprarenal fixation endografts for abdominal aortic aneurysms with neck length ≤10 mm in high-risk patients unfit for open repair and fenestrated endograft.* J Vasc Surg, 2016. **64**(3): p. 563-570.e1.
34. Gallitto, E., et al., *Endovascular Repair of Thoracoabdominal Aortic Aneurysm in High-Surgical Risk Patients: Fenestrated and Branched Endografts.* Ann Vasc Surg, 2017. **40**: p. 170-177.
35. Gerdes, C. and J.C. Nielsen, *Retrieval of Medtronic Micra Transcatheter Pacing System after tether removal.* Europace, 2016. **18**(8): p. 1202.
36. Gonsalves, S., et al., *Assessment of the efficacy of the rectovaginal button fistula plug for the treatment of ileal pouch-vaginal and rectovaginal fistulas.* Dis Colon Rectum, 2009. **52**(11): p. 1877-81.
37. Gratzke, C., et al., *Prostatic urethral lift vs transurethral resection of the prostate: 2-year results of the BPH6 prospective, multicentre, randomized study.* BJU Int, 2017. **119**(5): p. 767-775.
38. Greenberg, R.K., et al., *Intermediate results of a United States multicenter trial of fenestrated endograft repair for juxtarenal abdominal aortic aneurysms.* J Vasc Surg, 2009. **50**(4): p. 730-737.e1.

For peer review only

39. Grubman, E., et al., *To retrieve, or not to retrieve: System revisions with the Micra transcatheter pacemaker.* Heart Rhythm, 2017. **14**(12): p. 1801-1806.
40. Halak, M., M.A. Goodman, and S.R. Baker, *The fate of target visceral vessels after fenestrated endovascular aortic repair--general considerations and mid-term results.* Eur J Vasc Endovasc Surg, 2006. **32**(2): p. 124-8.
41. Harvey, J.R., et al., *Safety and feasibility of breast lesion localization using magnetic seeds (Magseed): a multi-centre, open-label cohort study.* Breast Cancer Res Treat, 2018. **169**(3): p. 531-536.
42. Hawasli, A., A. Phillips, and M. Tarboush, *Laparoscopic management of reflux after Roux-en-Y gastric bypass using the LINX system and repair of hiatal hernia: a case report.* Surg Obes Relat Dis, 2016. **12**(5): p. e51-e54.
43. Hawasli, A., M. Tarakji, and M. Tarboush, *Laparoscopic management of severe reflux after sleeve gastrectomy using the LINX(®) system: Technique and one year follow up case report.* Int J Surg Case Rep, 2017. **30**: p. 148-151.
44. Helmers, A.K., et al., *Comparison of the Battery Life of Nonrechargeable Generators for Deep Brain Stimulation.* Neuromodulation, 2018. **21**(6): p. 593-596.
45. Hemm, S., et al., *Contact position analysis of deep brain stimulation electrodes on post-operative CT images.* Acta Neurochir (Wien), 2009. **151**(7): p. 823-9; discussion 829.
46. Henderson, F., Jr. and I. Takacs, *Rapid and Minimally Traumatic Replacement of Stimulator Extension Cables: Technical Note on a Novel Use for Sternal Wire.* World Neurosurg, 2017. **97**: p. 547-550.
47. Herron, J.A., et al., *Chronic electrocorticography for sensing movement intention and closed-loop deep brain stimulation with wearable sensors in an essential tremor patient.* J Neurosurg, 2017. **127**(3): p. 580-587.
48. Holm, N., A. Müller, and R. Zbinden, *Complications with the MICRA TPS Pacemaker System: Persistent Complete Heart Block and Late Capture Failure.* Pacing Clin Electrophysiol, 2017. **40**(4): p. 455-456.
49. Iqbal, F.M., A. Bhatnagar, and R. Vidya, *Host Integration of an Acellular Dermal Matrix: Braxon Mesh in Breast Reconstruction.* Clin Breast Cancer, 2016. **16**(6): p. e209-e211.
50. Iqbal, M., et al., *Low power HOLEP after failed urolift: A case report using 50 Watt laser.* Urol Case Rep, 2018. **16**: p. 114-115.
51. Issa, K., et al., *Radiographic fit and fill analysis of a new second-generation proximally coated cementless stem compared to its predicate design.* J Arthroplasty, 2014. **29**(1): p. 192-8.
52. Jafferbhoy, S., et al., *Early multicentre experience of pre-pectoral implant based immediate breast reconstruction using Braxon(®).* Gland Surg, 2017. **6**(6): p. 682-688.
53. Kalder, J., et al., *Anatomic changes of target vessels after fenestrated and branched aortic aneurysm repair.* J Cardiovasc Surg (Torino), 2014. **55**(2 Suppl 1): p. 115-21.
54. Kaminska, M., et al., *Rechargeable deep brain stimulators in the management of paediatric dystonia: well tolerated with a low complication rate.* Stereotact Funct Neurosurg, 2012. **90**(4): p. 233-9.
55. Kaminska, M., et al., *Complications of Deep Brain Stimulation (DBS) for dystonia in children - The challenges and 10 year experience in a large paediatric cohort.* Eur J Paediatr Neurol, 2017. **21**(1): p. 168-175.
56. Kascak, P. and B. Kopcan, *Fatal Injury of the Small Intestine during Retropubic Sling Placement.* Case Rep Obstet Gynecol, 2015. **2015**: p. 164545.
57. Kawasaki, A., et al., *Comparing the risk of urethrolysis for the treatment of voiding dysfunction between two retropubic mesh slings: a case-control study.* Int Urogynecol J, 2013. **24**(4): p. 589-94.
58. Keehn, A., et al., *UroLift in Place of Fiducial Markers for Patients With Benign Prostatic Hyperplasia Undergoing External Beam Radiation Therapy.* Urology, 2017. **104**: p. 230-234.

For peer review only

59. Kelley, K. and J. Kim, *Human Factors Validation of the AeroForm Tissue Expander System for Breast Reconstruction.* Plast Surg Nurs, 2017. **37**(3): p. 100-102.
60. Kerwin, S.A., M.J. Mayotte, and C.C. Gornick, *Transcatheter pacemaker implantation in a patient with a bioprosthetic tricuspid valve.* J Interv Card Electrophysiol, 2015. **44**(1): p. 89-90.
61. Kitagawa, A., et al., *Zenith p-branch standard fenestrated endovascular graft for juxtarenal abdominal aortic aneurysms.* J Vasc Surg, 2013. **58**(2): p. 291-300.
62. Kypta, A., et al., *Dawn of a new era: the completely interventionally treated patient.* BMJ Case Rep, 2016. **2016**.
63. Lala, S., et al., *Superior mesenteric artery outcomes after fenestrated endovascular aortic aneurysm repair.* J Vasc Surg, 2016. **64**(3): p. 692-7.
64. Lin, J.-P., et al., *Bilateral globus pallidus internus deep brain stimulation for dyskinetic cerebral palsy supports success of cochlear implantation in a 5-year old ex-24 week preterm twin with absent cerebellar hemispheres.* European Journal of Paediatric Neurology, 2017. **21**(1): p. 202-213.
65. Lipham, J.C., et al., *The LINX® reflux management system: confirmed safety and efficacy now at 4 years.* Surg Endosc, 2012. **26**(10): p. 2944-9.
66. Lloyd, M., et al., *Rate adaptive pacing in an intracardiac pacemaker.* Heart Rhythm, 2017. **14**(2): p. 200-205.
67. Louie, B.E., et al., *Objective Evidence of Reflux Control After Magnetic Sphincter Augmentation: One Year Results From a Post Approval Study.* Ann Surg, 2019. **270**(2): p. 302-308.
68. Lyon, J.W., et al., *First experience with the use of a collagen fistula plug to treat enterocutaneous fistulas.* J Vasc Interv Radiol, 2013. **24**(10): p. 1559-65.
69. Madsen, A.M., et al., *A cohort study comparing a single-incision sling with a retropubic midurethral sling.* Int Urogynecol J, 2014. **25**(3): p. 351-8.
70. Maling, N., et al., *Biophysical basis of subthalamic local field potentials recorded from deep brain stimulation electrodes.* J Neurophysiol, 2018. **120**(4): p. 1932-1944.
71. Maruccia, M., G. Di Taranto, and M.G. Onesti, *One-stage muscle-sparing breast reconstruction in elderly patients: A new tool for retaining excellent quality of life.* Breast J, 2018. **24**(2): p. 180-183.
72. Maruccia, M., et al., *One-stage breast reconstruction techniques in elderly patients to preserve quality of life.* Eur Rev Med Pharmacol Sci, 2016. **20**(24): p. 5058-5066.
73. McAdams, S., et al., *Holmium Laser Enucleation of the Prostate After Prostatic Urethral Lift Surgery: Feasibility and Technical Considerations from a Multi-Institutional Case Series.* J Endourol, 2017. **31**(8): p. 774-779.
74. McNicholas, T.A., et al., *Minimally invasive prostatic urethral lift: surgical technique and multinational experience.* Eur Urol, 2013. **64**(2): p. 292-9.
75. Morita, J., et al., *Retrieval of a Micra transcatheter pacing system in a heart with a preexisting lead.* Indian Pacing Electrophysiol J, 2018. **18**(5): p. 183-184.
76. Nam, D., et al., *An evaluation of proximal femur bone density in young, active patients undergoing total hip arthroplasty at one year postoperatively.* Hip international : the journal of clinical and experimental research on hip pathology and therapy, 2019. **29**(1): p. 51-57.
77. Neumann, W.J., et al., *Long term correlation of subthalamic beta band activity with motor impairment in patients with Parkinson's disease.* Clin Neurophysiol, 2017. **128**(11): p. 2286-2291.
78. Niemann, M., et al., *Longevity of Implantable Pulse Generators in Bilateral Deep Brain Stimulation for Movement Disorders.* Neuromodulation, 2018. **21**(6): p. 597-603.
79. Ondo, W.G., C. Meilak, and K.D. Vuong, *Predictors of battery life for the Activa Soletra 7426 Neurostimulator.* Parkinsonism Relat Disord, 2007. **13**(4): p. 240-2.

For peer review only

80. Onesti, M.G., et al., *Clinical, histological, and ultrasound follow-up of breast reconstruction with one-stage muscle-sparing "wrap" technique: A single-center experience.* J Plast Reconstr Aesthet Surg, 2017. **70**(11): p. 1527-1536.
81. O'Rawe, J.A., et al., *Integrating precision medicine in the study and clinical treatment of a severely mentally ill person.* PeerJ, 2013. **1**: p. e177.
82. Parmar, A.D., et al., *Two-Stage Explantation of a Magnetic Lower Esophageal Sphincter Augmentation Device Due to Esophageal Erosion.* J Laparoendosc Adv Surg Tech A, 2017. **27**(8): p. 829-833.
83. Perez, J., et al., *Rechargeable or Nonrechargeable Deep Brain Stimulation in Dystonia: A Cost Analysis.* Neuromodulation, 2017. **20**(3): p. 243-247.
84. Piccini, J.P., et al., *Long-term outcomes in leadless Micra transcatheter pacemakers with elevated thresholds at implantation: Results from the Micra Transcatheter Pacing System Global Clinical Trial.* Heart Rhythm, 2017. **14**(5): p. 685-691.
85. Pollo, C., et al., *Magnetic resonance artifact induced by the electrode Activa 3389: an in vitro and in vivo study.* Acta Neurochir (Wien), 2004. **146**(2): p. 161-4.
86. Price, E.R., et al., *Initial Clinical Experience With an Inducible Magnetic Seed System for Preoperative Breast Lesion Localization.* AJR Am J Roentgenol, 2018. **210**(4): p. 913-917.
87. Quinn, E.J., et al., *Beta oscillations in freely moving Parkinson's subjects are attenuated during deep brain stimulation.* Mov Disord, 2015. **30**(13): p. 1750-8.
88. Ramirez de Noriega, F., et al., *Constant Current versus Constant Voltage Subthalamic Nucleus Deep Brain Stimulation in Parkinson's Disease.* Stereotact Funct Neurosurg, 2015. **93**(2): p. 114-121.
89. Reynolds, D., et al., *A Leadless Intracardiac Transcatheter Pacing System.* N Engl J Med, 2016. **374**(6): p. 533-41.
90. Reynolds, J.L., et al., *Magnetic sphincter augmentation with the LINX device for gastroesophageal reflux disease after U.S. Food and Drug Administration approval.* Am Surg, 2014. **80**(10): p. 1034-8.
91. Reynolds, J.L., et al., *Laparoscopic Magnetic Sphincter Augmentation vs Laparoscopic Nissen Fundoplication: A Matched-Pair Analysis of 100 Patients.* J Am Coll Surg, 2015. **221**(1): p. 123-8.
92. Ritter, P., et al., *Early performance of a miniaturized leadless cardiac pacemaker: the Micra Transcatheter Pacing Study.* Eur Heart J, 2015. **36**(37): p. 2510-9.
93. Roberts, P.R., et al., *A leadless pacemaker in the real-world setting: The Micra Transcatheter Pacing System Post-Approval Registry.* Heart Rhythm, 2017. **14**(9): p. 1375-1379.
94. Roehrborn, C.G., et al., *Five year results of the prospective randomized controlled prostatic urethral L.I.F.T. study.* Can J Urol, 2017. **24**(3): p. 8802-8813.
95. Roehrborn, C.G., et al., *Three year results of the prostatic urethral L.I.F.T. study.* Can J Urol, 2015. **22**(3): p. 7772-82.
96. Rona, K.A., et al., *Efficacy of magnetic sphincter augmentation in patients with large hiatal hernias.* Surg Endosc, 2017. **31**(5): p. 2096-2102.
97. Rona, K.A., et al., *Hiatal hernia recurrence following magnetic sphincter augmentation and posterior cruroplasty: intermediate-term outcomes.* Surg Endosc, 2018. **32**(7): p. 3374-3379.
98. Rukstalis, D., et al., *24-month durability after crossover to the prostatic urethral lift from randomised, blinded sham.* BJU Int, 2016. **118 Suppl 3**: p. 14-22.
99. Salvador, R., et al., *Esophageal Penetration of the Magnetic Sphincter Augmentation Device: History Repeats Itself.* J Laparoendosc Adv Surg Tech A, 2017. **27**(8): p. 834-838.
100. Sammartino, F., et al., *3-Tesla MRI in patients with fully implanted deep brain stimulation devices: a preliminary study in 10 patients.* J Neurosurg, 2017. **127**(4): p. 892-898.
101. Shahverdyan, R., et al., *Technical feasibility of endovascular aortoiliac aneurysm repair combining Anaconda fenestrated and Zenith iliac side-branched stent grafts.* J Vasc Surg, 2015. **61**(5): p. 1324-8.

For peer review only

102. Shahverdyan, R., et al., *F-EVAR does not Impair Renal Function more than Open Surgery for Juxtarenal Aortic Aneurysms: Single Centre Results.* Eur J Vasc Endovasc Surg, 2015. **50**(4): p. 432-41.
103. Sheu, E.G., et al., *A comparative trial of laparoscopic magnetic sphincter augmentation and Nissen fundoplication.* Surg Endosc, 2015. **29**(3): p. 505-9.
104. Shute, J.B., et al., *Thalamocortical network activity enables chronic tic detection in humans with Tourette syndrome.* Neuroimage Clin, 2016. **12**: p. 165-72.
105. Soejima, K., et al., *Performance of Leadless Pacemaker in Japanese Patients vs. Rest of the World - Results From a Global Clinical Trial.* Circ J, 2017. **81**(11): p. 1589-1595.
106. Soejima, K., et al., *Safety evaluation of a leadless transcatheter pacemaker for magnetic resonance imaging use.* Heart Rhythm, 2016. **13**(10): p. 2056-63.
107. Sriratanaviriyakul, N., et al., *LINX®, a novel treatment for patients with refractory asthma complicated by gastroesophageal reflux disease: a case report.* J Med Case Rep, 2016. **10**(1): p. 124.
108. Starnes, B.W., *Physician-modified endovascular grafts for the treatment of elective, symptomatic, or ruptured juxtarenal aortic aneurysms.* J Vasc Surg, 2012. **56**(3): p. 601-7.
109. Swann, N.C., et al., *Chronic multisite brain recordings from a totally implantable bidirectional neural interface: experience in 5 patients with Parkinson's disease.* J Neurosurg, 2018. **128**(2): p. 605-616.
110. Swann, N.C., et al., *Adaptive deep brain stimulation for Parkinson's disease using motor cortex sensing.* J Neural Eng, 2018. **15**(4): p. 046006.
111. Syrkin-Nikolau, J., et al., *Subthalamic neural entropy is a feature of freezing of gait in freely moving people with Parkinson's disease.* Neurobiol Dis, 2017. **108**: p. 288-297.
112. Tapping, C.R., et al., *Three-year follow-up of fenestrated thoracoabdominal stent graft bridging an endovascular thoracic stent graft and a surgical abdominal aortic graft.* J Vasc Interv Radiol, 2011. **22**(3): p. 385-90.
113. Tapping, C.R., M.W. Little, and P. Boardman, *Successful Prostatic Artery Embolization following UroLift Device Failure.* J Vasc Interv Radiol, 2017. **28**(9): p. 1275-1276.
114. Tatum, J.M., et al., *Minimal versus obligatory dissection of the diaphragmatic hiatus during magnetic sphincter augmentation surgery.* Surg Endosc, 2019. **33**(3): p. 782-788.
115. Timaran, D.E., et al., *Fenestrated endovascular aneurysm repair among octogenarians at high and standard risk for open repair.* J Vasc Surg, 2017. **66**(2): p. 354-359.
116. Timaran, D.E., et al., *Safety and effectiveness of total percutaneous access for fenestrated endovascular aortic aneurysm repair.* J Vasc Surg, 2016. **64**(4): p. 896-901.
117. Trager, M.H., et al., *Subthalamic beta oscillations are attenuated after withdrawal of chronic high frequency neurostimulation in Parkinson's disease.* Neurobiol Dis, 2016. **96**: p. 22-30.
118. Ugurlucan, F.G., et al., *Randomized trial of graft materials in transobturator tape operation: biological versus synthetic.* Int Urogynecol J, 2013. **24**(8): p. 1315-23.
119. Ultee, K.H.J., et al., *Perioperative outcome of endovascular repair for complex abdominal aortic aneurysms.* J Vasc Surg, 2017. **65**(6): p. 1567-1575.
120. Van Gompel, J.J., et al., *Anterior nuclear deep brain stimulation guided by concordant hippocampal recording.* Neurosurg Focus, 2015. **38**(6): p. E9.
121. Vemuri, C., et al., *Postapproval outcomes of juxtarenal aortic aneurysms treated with the Zenith fenestrated endovascular graft.* J Vasc Surg, 2014. **60**(2): p. 295-300.
122. Vidya, R. and S.J. Cawthorn, *Muscle-Sparing ADM-Assisted Breast Reconstruction Technique Using Complete Breast Implant Coverage: A Dual-Institute UK-Based Experience.* Breast Care (Basel), 2017. **12**(4): p. 251-254.
123. Vidya, R., et al., *Evaluation of the effectiveness of the prepectoral breast reconstruction with Braxon dermal matrix: First multicenter European report on 100 cases.* Breast J, 2017. **23**(6): p. 670-676.

For peer review only

124. Waln, O. and J. Jimenez-Shahed, *Rechargeable deep brain stimulation implantable pulse generators in movement disorders: patient satisfaction and conversion parameters.* Neuromodulation, 2014. **17**(5): p. 425-30; discussion 430.
125. Wang, S.K., et al., *Successful Treatment of an Infected Zenith Fenestrated Endograft Without Explantation.* Vasc Endovascular Surg, 2018. **52**(7): p. 569-572.
126. Warren, H.F., et al., *Factors influencing the outcome of magnetic sphincter augmentation for chronic gastroesophageal reflux disease.* Surg Endosc, 2018. **32**(1): p. 405-412.
127. Wooster, M., et al., *Concomitant Parallel Endografting and Fenestrated Experience in a Regional Aortic Center.* Ann Vasc Surg, 2017. **38**: p. 54-58.
128. Zeidler, K.R., et al., *AeroForm patient controlled tissue expansion and saline tissue expansion for breast reconstruction: a randomized controlled trial.* Ann Plast Surg, 2014. **72 Suppl 1**: p. S51-5.

For peer review only
